# The Developmental Origins of Joint Attention: Infants' Early Joint Attention Bids

**DOI:** 10.1111/infa.70012

**Published:** 2025-03-26

**Authors:** Gideon Salter, Malinda Carpenter

**Affiliations:** ^1^ School of Psychology and Neuroscience University of St Andrews St Andrews UK; ^2^ Department of Psychology University of York York UK

**Keywords:** communication, infant development, joint attention, longitudinal data, social cognition

## Abstract

There are theoretical debates about the definition of joint attention, and empirical debates about when it emerges in development. Here we addressed both debates by investigating the emergence of infants' communicative joint attention bids: looks to their partner's face, accompanied by communicative facial expressions and/or vocalizations, to attempt to initiate joint attention to a referent. We tested 25 infants monthly, longitudinally, between 6 and 10 months using both novel joint attention elicitation tests and free play observations. Even when using a conservative definition of joint attention involving communication, results indicated that a substantial percentage of infants (44%) had already begun to produce joint attention bids by 6 months, with the vast majority (92%) having done so before 9 months. Joint attention bids emerged gradually, with increasing consistency, and were seen earlier in the novel elicitation tests than in free play, suggesting that previous work focusing on free play might have underestimated infants' joint attention. We discuss the implications of these findings for theories of joint attention and communication.

## Introduction

Despite longstanding recognition of the importance of joint attention in human social and cultural development (e.g., Carpendale and Lewis 2006; Mundy and Newell 2007; Reddy 2010; Tomasello 1995, 2019), there are still theoretical and empirical debates about its definition and development. Theoretically, there are debates about how the jointness in joint attention is achieved. One key task is to distinguish situations in which two agents are merely individually attending to a common locus from situations in which both co‐attenders are aware that they are sharing attention (R. P. Hobson 2005; Tomasello 1995). Some have argued that to truly achieve this jointness—this sharedness and mutuality—there needs to be active communication (Carpenter and Liebal 2011; Eilan, n.d.; Siposova and Carpenter 2019). Intentionally communicative signals make public one's attention to a feature of the world, and are produced in order to draw another's attention to that same feature, and/or confirm that both attenders are attending to that feature and/or comment on it (Breheny 2006; Moore 2017; Sperber and Wilson 1995). It has been suggested that one of the simplest ways in which infants can engage in communicative joint attention, earlier than behaviors like showing or pointing, is via a communicative look (Bruner 1975; Carpenter and Liebal 2011). This is eye contact with one's partner coordinated with a facial expression, in infancy typically a smile, as a communicative comment on some feature of the world (J. A. Hobson and Hobson 2007; Jones and Hong 2001; Striano and Bertin 2005; Venezia et al. 2004).

There have been some prior attempts to operationally define how a look can be joint or shared. J. A. Hobson and Hobson (2007) sought to operationalize “sharing looks” using subjective judgments about whether these looks were characterized by personal involvement and emotional engagement, rather than merely checking that the other is present. However, Graham et al. (2021) found that this coding strategy was insufficiently specific for the purpose of behavioral coding, with raters failing to achieve reliability when applying Hobson and Hobson's scheme. Graham and colleagues thus argued for an approach to the analysis of joint attention that focuses on clearly identifiable behaviors, rather than broad subjective judgments.

There are some previous studies that have examined such clearly identifiable behaviors. Jones and colleagues (Jones et al. 1991; Jones and Hong 2001) and Venezia‐Parlade and colleagues (Venezia et al. 2004; Venezia‐Parlade et al. 2009) have examined infants' “anticipatory smiles,” in which the onset of infants' smiles begins prior to their look toward a caregiver. The temporal ordering of smiling and then looking to the caregiver indicates that the smile is not simply a response to the caregiver's face, but rather a spontaneous act by the infant. Jones and Hong (2001) found that this behavior sequence was observed in 8‐month‐old infants (the youngest age they tested), and relations were identified between anticipatory smiles and intentionally communicative gestures and vocalizations, leading the authors to describe anticipatory smiling as the “onset of voluntary communication” (p. 353). Furthermore, Striano and Bertin (2005), though not focusing on early communication, identified “joint engagement looks with smile,” which they defined as infants looking from a toy to the interaction partner's face and back again, with a spontaneous smile produced during the look to the interaction partner. They identified some rare cases at 5 months (4% of infants) and 7 months (13% of infants), and found a gradual emergence of “joint engagement looks with smile” between 5 and 9 months.

Prior work has focused on smiles, but it is also important to recognize that communicative looks can be accompanied by other types of facial expressions (e.g., frowns, quizzical expressions) and/or vocalizations (Donnellan et al. 2020; Gros‐Louis et al. 2014; Messinger 2002). Therefore, borrowing a term from Mundy (1995), we use “joint attention bids” to describe communicative looks to a partner's face, accompanied by facial expressions and/or vocalizations, that attempt to initiate joint attention to a referent.

Along with theoretical debates about how joint attention is achieved, empirically, there are also debates about when joint attention first emerges in development. Some researchers have argued that joint attention emerges suddenly at around 9 months of age (e.g., Hubley and Trevarthen 1979; Stern 1985; Tomasello 1999), as evidenced by the emergence of gaze alternation between an object and an interaction partner, and production of early declarative gestures such as showing and pointing (e.g., E. Bates et al. 1979; Cameron‐Faulkner et al. 2015; Carpenter et al. 1998). However, this perspective has been criticized on a number of fronts, with some researchers instead claiming a more gradual emergence of joint attention beginning earlier in infants' first year (de Barbaro et al. 2013; de Barbaro et al. 2016; Hoehl and Striano 2013; Moll et al. 2021; Reddy 2010; Rossmanith et al. 2014; Striano and Bertin 2005).

Conclusions about when joint attention emerges depend on the definitions and the methods used, meaning that these theoretical and empirical issues are intertwined. Both the definitions of joint attention and the methods used have diverged across different studies. For example, in contrast to classic studies that focused on gaze alternation and/or declarative gestures (e.g., Bakeman and Adamson 1984; Carpenter et al. 1998; Mundy et al. 2003), Rossmanith et al. (2014) coded situations in which infants responded to caregivers directing their attention as instances of joint attention, and de Barbaro et al. (2013; see also 2016) focused on infants' and caregivers' simultaneous visual attention to and manual activity on objects. Though these are relevant forms of social engagement, they do not provide evidence for the emergence of infants' capacity to actively try to initiate joint attention themselves, or, necessarily, infants' awareness of the jointness of the engagement. Striano and Bertin (2005) focused on infants' coordination of smiles and looks, but so far, no previous studies in this age range have focused on the communicative use of these acts to try to initiate joint attention.

Similarly, in terms of the methods used, although most of the classic studies measured joint attention during infant free play with a caregiver, there are some studies that were designed to elicit joint attention (e.g., Mundy et al. 2003; Perucchini and Camaioni 1993). However, these have task features that may make them challenging for young infants, and thus they might underestimate infants' capabilities. For example, the “Object Spectacle Tasks” that form part of the Early Social Communication Scales (ESCS; Mundy et al. 2003) involve activating wind‐up toys to give participants an interesting sight about which to initiate communication/joint attention. In these previous studies, the stimuli move continuously (often additionally making noise) throughout the response period, presenting participants with a very attention‐grabbing stimulus that might be cognitively demanding for young infants to look away from. In addition, the fact that the infant has witnessed the adult activate the stimulus means that the object is arguably already being attended to by both the infant and the adult, thus potentially reducing the likelihood of a communicative initiation by the infant, and making the extent to which the infant is taking an initiating role more ambiguous.

These differences in definitions and methods make it difficult to compare findings across studies and come up with a good picture of the development of joint attention. In the current study, we combined the strengths of some of the previous different approaches to joint attention in the literature and used both (1) a conservative definition that captures the key feature of jointness in joint attention and, at the same time, (2) a method that allows us to identify infants' earliest spontaneous attempts to bid for joint attention. We thus provide monthly longitudinal data from 6‐ to 10‐month‐old infants with the aim of capturing the very beginnings of joint attention. We employed a conservative definition that requires, for the strongest indications of joint attention bids, active communication, here in its simplest form: a communicative look about the stimulus (e.g., Carpenter and Liebal 2011; Eilan, n.d.). We tested infants both in the classic free play situation with their mother, and also in three novel joint attention bid elicitation tests that adapt previous procedures to optimize young infants' chances of initiating communicative joint attention bids. These tasks involved repeatedly activating some stimulus (interesting sight, interesting sound, or moving object) out of sight of an experimenter (E) but in sight of infants, to give infants the opportunity to attempt to share that stimulus with E through joint attention bids (similarly to how declarative point elicitation tasks, e.g. Liszkowski et al. 2004, elicit attempts to share attention with an experimenter who is not currently looking at the stimulus). We also provide a new coding approach that assesses whether infants' looks are communicative behaviors that involve active sharing of the stimulus. In addition to this conservative approach, we also coded for non‐communicative looks, to investigate whether there were non‐communicative precursors to joint attention bids (or evidence of “mutual” along with “shared” attention in Siposova and Carpenter's 2019, terms).

This study makes a number of contributions to both the theoretical and empirical study of both joint attention and communication. It contributes needed information to several active theoretical debates in psychology and philosophy, including debates about how the jointness in joint attention is achieved, and the cognitive mechanisms required for communication. It is the first study that directly investigates the early development of joint attention bids using a conservative coding scheme that focuses on observable communicative behaviors rather than subjective impressions of “sharing” (Graham et al. 2021). In doing so, it examines the very beginnings of both joint attention and communication. The study introduces a novel elicitation method that was designed to strip away caregiver scaffolding and distractions that are present in naturalistic interactions, enabling clear assessment of infants' own capabilities. It also makes use of varied stimuli, examining joint attention to auditory stimuli as well as visual stimuli, an area that has recently been highlighted as an underexplored area of investigation in studies of joint attention (e.g., Adamson et al. 2021; Battich et al. 2020; Gabouer and Bortfeld 2021).

## Method

### Participants

Twenty‐five mother‐infant dyads participated (14 female infants, 11 male infants; 14 firstborns; all full‐term). Infants were recruited in St Andrews, Scotland, and the surrounding area through online advertisements, in‐person connections at local groups and activities, and through an education and support group that met in the University of St Andrews Baby and Child Lab (see Salter et al. 2021). Information about parents' educational background was provided by the mothers of 23 of the infants. Of these, 20 had at least one parent who had completed tertiary education, and 3 had at least one parent who had completed secondary education. No further data on socio‐economic circumstances were collected directly from participants, but data from a national index of relative deprivation that takes into account a range of information on health, unemployment and income to identify the relative deprivation of different regions in the country (Scottish Government 2020) indicated local variability in socio‐economic circumstances, with local areas ranging from the fourth to the 10th decile (where the 10th decile includes the least deprived areas in the country). The sample size was determined in advance of testing based on previous similar longitudinal studies using close coding of infant joint attention (e.g., Bakeman and Adamson 1984; Carpenter et al. 1998; see also, e.g., Beuker et al. 2013; Northrup and Iverson 2020, for recent comparable studies). Sessions were conducted in the lab within a week of infants' monthly birthdays, longitudinally once a month from 6 to 10 months inclusive. Only one infant missed a single session (at 10 months), meaning that there were 124 sessions in total. The present study was conducted according to guidelines laid down in the Declaration of Helsinki, with written informed consent obtained from a parent or guardian for each child before any assessment or data collection. All procedures involving human subjects in this study were approved by both the School of Psychology and Neuroscience Ethics Committee and the University Teaching and Research Ethics Committee at the University of St Andrews.

### Procedure

The tasks were conducted as part of a larger study on the development of joint attention and communication and other skills. Each session began with a mother‐infant free play period, followed by a number of different tests that examined a range of social and cognitive abilities (e.g., communicative gestures, attention following, imitation, object permanence). The free play period was always administered first to reduce the need to rearrange the testing space repeatedly. The three joint attention elicitation tests were interspersed randomly amongst the other tests. The entire procedure was video‐recorded with two cameras from different angles. For an overview of the full longitudinal study procedure, see the doctoral thesis in which the full study is described (Salter 2022). Photos of some of the study materials and the testing setup can be found in the Supplementary Materials (SM1).

#### Free Play

Mothers and infants first participated in a 6 min free play period with toys (e.g., rattles, rubber bath toys, board books); 6 min was chosen to provide an adequate amount of time for observation while also keeping the testing session short enough for young infants. They sat on a padded mat on the floor. If infants were unable to sit independently, a Bumbo support seat was provided for the first 3 min to ensure that in all sessions infants spent at least some of the time in a sitting position. After this time had elapsed, mothers were asked to place infants in whatever position was comfortable for them. Mothers were asked to play as they typically would at home, with whichever toys they wanted to use.

#### Joint Attention Elicitation Tests

In the joint attention elicitation tests, we varied the types of stimuli that served as the target of joint attention: an interesting stationary sight, a moving toy, and a sound. Together, the three tests lasted around 3 min total. Infants sat upright in an infant chair opposite E, who sat on the floor, such that infants' and E's eyes were at approximately the same level. Mothers sat in an oblique position with respect to the infant's gaze, behind and to the right of E, so that they remained in view of infants but were not facing directly toward them. They were asked to refrain from interacting with infants as much as possible during the tests, and were occupied during the tests by filling out questionnaires or reading a magazine.

##### Interesting Sight Test

For this test, the stimulus was a set of white LED lights placed inside a translucent blue plastic box, activated by remote control. A different, randomized pattern of flashing was used at each session. A child‐sized table was positioned on the left‐hand side of E. E gave infants a small toy as a distraction, before placing an occluding barrier on the table. E then placed the lights on the table, with the barrier obscuring infants' view of the lights. E then took away the toy from infants, before quickly removing the occluder while simultaneously surreptitiously activating the lights with a remote control held hidden behind his back. E then sat facing infants, with the remote control still held hidden. E activated the lights three times in total per task. When the lights were on, E waited until infants looked at the lights and then left the lights on for 5 s. This was to ensure that infants had seen the stimulus. The lights were then turned off for 5 s. This was done to make it easier for infants to disengage their attention from the stimulus to look at E. If at any point infants made eye contact with E, E said, “What?”, “What is it?” or “What is it, [infant's name]?” in an inquisitive tone, without ever turning away from infants' face to look at the stimulus. The purpose of this type of response was for E to act as a willing interaction partner without displaying any awareness of the stimulus. This approach is similar to the approach used by Perucchini and Camaioni (1993) to elicit declarative pointing, and by Striano and Rochat (2000) to elicit joint attention. However, in this procedure E displayed no positive affect and did not ever turn to look at the stimulus, to limit the sense that E was aware of the stimulus, encourage further communicative behaviors from infants, and reduce the likelihood that infants were solely smiling in response to E. E's face remained expressionless when he was not responding to infants, and if infants did not look to E at any point then E remained expressionless and did not speak. After completing three cycles of activation and deactivation, E removed the stimulus from the table and placed it back behind him.

##### Moving Toy Test

Except for the stimuli, the procedure of the Moving Toy test was almost identical to that of the Interesting Sight test. Stimuli for this test were various remote‐controlled toys (e.g., a moving toy robot). Each made different noises and had different patterns of flashing lights. E activated the toy 3 times per task, again for 5 s once infants had seen it, with a 5 s pause between activations. All objects moved forwards and backwards twice per activation, except the turtle which rotated in place. One slight difference in the Moving Toy test compared to the Interesting Sight test was that E only responded to infants while the toy was not active, to ensure that he was not speaking while the toy was producing sound.

##### Interesting Sound Test

Stimuli for this test were different objects that made a noise (e.g., a xylophone). The child‐sized table was placed between infants and E. E waited until infants were looking away from E or, if they were unsettled, E gave them a small toy as a distraction. Once infants were distracted, the procedure was similar to the Interesting Sight and Moving Toy tests. While looking at infants, E surreptitiously activated the stimulus three times per test, with the stimulus located in front of E and the table obscuring the sight of the stimulus for infants. Again, there was a 5 s pause between activations. During each activation, the stimulus made the noise three times (e.g., the xylophone was struck 3 times). E responded to any looks to his face as above, though again he did not speak over the sound being produced. Care was taken to prevent infants from seeing any of E's movements, and E did not look down at the stimulus during the procedure. At the end of the procedure, E revealed the stimulus to infants to make the procedure similar to the Interesting Sight and Moving Toy procedures, in which E removed the target object at the end of the procedure.

A video of a joint attention bid produced by a 6‐month‐old infant to a moving toy stimulus can be viewed on an Open Science Foundation repository (https://osf.io/z52ns).

### Coding

All four tasks were scored using the same three‐level coding scheme. A general overview of the coding scheme can be found in Table 1, and a full version of the coding scheme can be found in SM2. A score of “2” was given for a spontaneous joint attention bid, that is, a communicative look to the face of E or infants' mother (M) that was about a toy (in the free play) or the stimulus (in the tests). A score of “1” was given for a spontaneous but non‐communicative look to the face of E or M that was about the toy/stimulus. A score of “0” was given if infants did not look to the face of E or M, or did so only in response to their behavior. For each elicitation test and the free play, infants received a single score of “0”, “1” or “2” for each monthly session (i.e., the highest score they had received for that test at that session). This was because our focus was not on the number of looks produced in each test, but rather on whether or not a joint attention bid was produced. For the elicitation tests, infants' mothers were always present, though not involved in the procedure. We did not want to ignore infants' looks to them; thus, in the elicitation tests, if the look was to their mother, infants could still receive a score for a joint attention bid.

**TABLE 1:** Summary of coding scheme for joint attention bids.

Score	Description
2	Infants a) looked from the toy/stimulus to the face of E or M, b) produced a distinct facial expression or vocalization that was coordinated concurrently with the look as a comment on the toy/stimulus, c) produced these behaviors to communicate about a clear referent, and d) did not do so merely in response to E or M.
1	Infants looked from the toy/stimulus to the face of E or M. The look was about the toy/stimulus and was not merely in response to E or M. However, the look was not a communicative look, that is, it was not coordinated with a distinct facial expression or communicative vocalization (infants had a neutral facial expression).
0	Infants did not look from the toy/stimulus to the face of E or M, looking only at the toy/stimulus or elsewhere. Or if infants did look to the face of E or M, this was only in response to some behavior by E or M, or the look was not clearly about or contingent upon the toy/stimulus.

There are several ways in which this coding scheme differs from previous approaches. First, coding schemes that focus on gaze alternation typically require a look from a stimulus to an agent and back to the stimulus (e.g., Bakeman and Adamson 1984). Here, we required only a look from the stimulus to the adult, without requiring a look back to the stimulus. This was because our focus was on the behavior accompanying the look to the adult as the key evidence of communicativeness. Additionally, the design of the elicitation tests made requiring a look back to the stimulus problematic, as infants' attention would likely be drawn to the stimuli once they reactivated, making it challenging to establish why infants performed this look. However, because of the widespread use of this requirement in research on joint attention, we also examined whether there were looks back to the toy/stimulus in those tests in which a visible toy/stimulus was used (Interesting Sight, Moving Toy, Free Play).

Second, there were two types of behavior that could serve as evidence for the communicativeness of the look: facial expressions and vocalizations. The majority of previous work has focused on smiles, and for good reason: joint attention episodes often involve positive affect (Leavens et al. 2014; Messinger and Fogel 2007). Since our focus was not solely on the sharing of positive affect, but instead on communicativeness, we also allowed for other facial expressions (e.g., frowns) that were expressive and about the toy/stimulus, and vocalizations (Donnellan et al. 2020; Messinger 2002). The vocalizations had to be distinct (not already occurring prior to the look), about the toy/stimulus, and not vegetative.

All sessions were coded by the first author. An independent coder who was unaware of the hypotheses of the study coded 100% of the elicitation tests, and a randomly‐chosen 33% of the free play sessions. A Cohen's Kappa of 0.71 was obtained across tasks, with 73% agreement (for further information see SM3). In all cases but one, the difference was of one score (i.e., between “0” and “1”, or “1” and “2”).

## Results

Generalized linear mixed‐effects models (GLMMs) were fit to the data using the package lme4 (D. Bates et al. 2015). Each model used a binomial error structure and logit link function. Post hoc comparisons were conducted using emmeans (Lenth 2024). Further information about the analyses and packages used, including tables with model coefficients, can be found in SM4, SM5, and SM6. Pseudonymised data and the analysis script in R Markdown format are available as part of the Supplementary Material (SM7 to SM11; see also https://osf.io/z52ns/).

### Emergence of Joint Attention Bids

**FIGURE 1:**
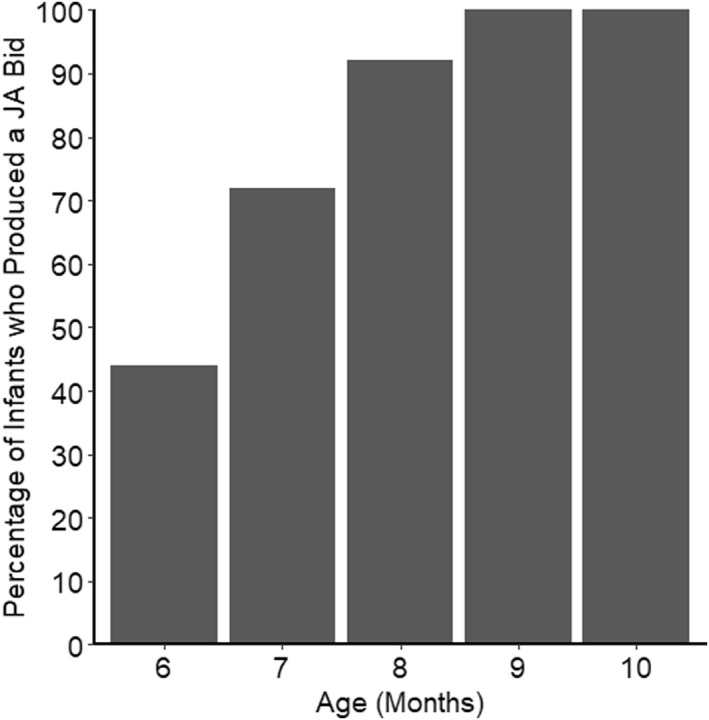
Percentage of Infants who had Produced a Joint Attention Bid by each Age (Cumulatively) Across all Three Tests and Free Play Combined.

The first set of analyses focused on the emergence of infants' earliest joint attention bid (i.e., score of “2”), using scores combined across all three elicitation tests and free play. Figure 1 displays the percentage of infants who had produced a joint attention bid by each age.

At the first session, when participants were 6 months of age, 11 out of 25 infants (44%) produced at least one joint attention bid, and by 7 months, 18 out of 25 (72%) had done so. By 9 months, all participants had produced at least one joint attention bid. In all cases in which a facial expression was produced, it was a smile. For the free play and the elicitation tests which involved a stimulus clearly located in space (i.e., all tests except the Interesting Sound test), we examined the percentage of cases in which infants' first gaze shift after producing the joint attention bid was a look back to the object. Overall, joint attention bids were followed by a look back to the object in the majority (82%) of cases (Interesting Sight: 85%, Moving Toy: 91%, Free Play: 65%). For further information, see SM5.2.

**FIGURE 2:**
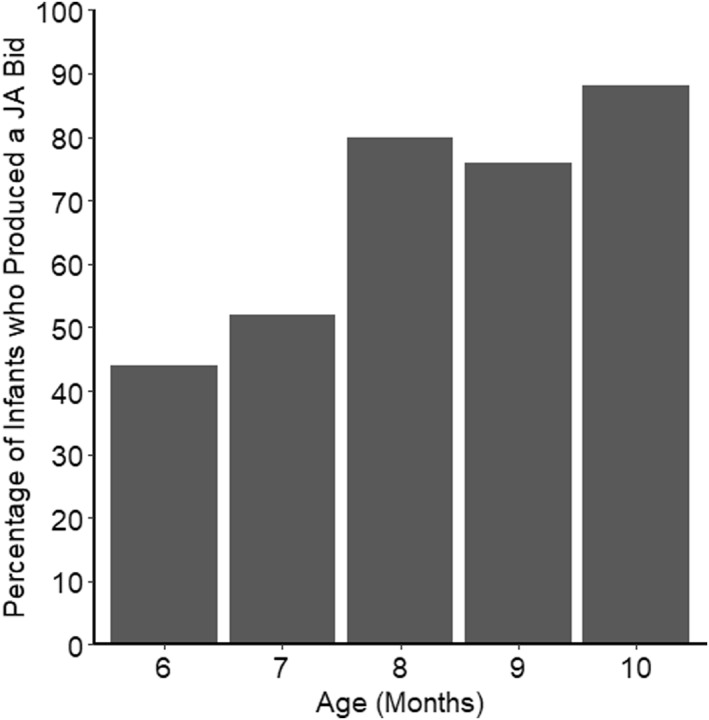
Percentage of Infants who Produced a Joint Attention Bid at each Age Across all Three Tests and Free Play Combined.

Figure 2 shows the percentage of infants who produced a joint attention bid at each age (rather than the cumulative percentage who had passed by each age, as in Figure 1). It indicates that it was not always the case that once infants had produced a joint attention bid, they continued to do so at every session.

To investigate developmental changes in the number of infants who produced joint attention bids, GLMMs were specified. The dependent variable was a binomial outcome, Joint Attention Bid Production (0 for no joint attention bid in any task, i.e., a score of “0” or “1” in all tasks; 1 for a joint attention bid, i.e., a score of “2” in at least one task). In the first model, Age (centered) was a continuous fixed effect and Participant was a random effect. Age (centered) was a significant predictor of Joint Attention Bid Production in this model (log odds_baseline_ = 0.92[0.44,1.54], *p* < 0.001; log odds_age_ = 0.02[0.01,0.03], *p* < 0.001), with joint attention bids becoming increasingly likely as children aged. Next, we examined whether there were any rapid changes in joint attention bid production, indexed by a significant change between consecutive months. In a second GLMM, Age was fit as a categorical fixed effect to enable comparisons between scores at each pair of months and to capture any potential rapid shifts in joint attention bid production, with Participant as a random effect. Tukey's HSD post hoc tests on Age revealed significant increases in infants' production of joint attention bids only between 6 and 10 months (log odds = 2.34[0.22,4.47], *p* = 0.022), though the lower bound of the 95% confidence intervals for the effect size was below the threshold of a small effect size (log odds<0.36). There was no significant difference between any two consecutive months. Similar results were found when the free play and elicitation tests (combined) were analyzed separately (see SM5 for details).

In summary, these results show that a substantial percentage (44%) of infants produced at least one joint attention bid at 6 months, with almost all infants (23 out of 25; 92%) having produced at least one joint attention bid by 8 months. Although we did not make a look back to the stimulus a requirement for our definition of a joint attention bid, a look back occurred in a substantial majority of cases (83%; see SM2.1 for details on the coding of looks back, and Figure S5.3, SM5 for more information). Joint attention bid production increased with age, but no rapid changes in joint attention bid production between consecutive months were detected. However, these results ought to be interpreted with some caution given the limitations of the sample size to detect small differences between consecutive months.

### Comparing Elicitation Test Scores With Free Play Scores

**FIGURE 3:**
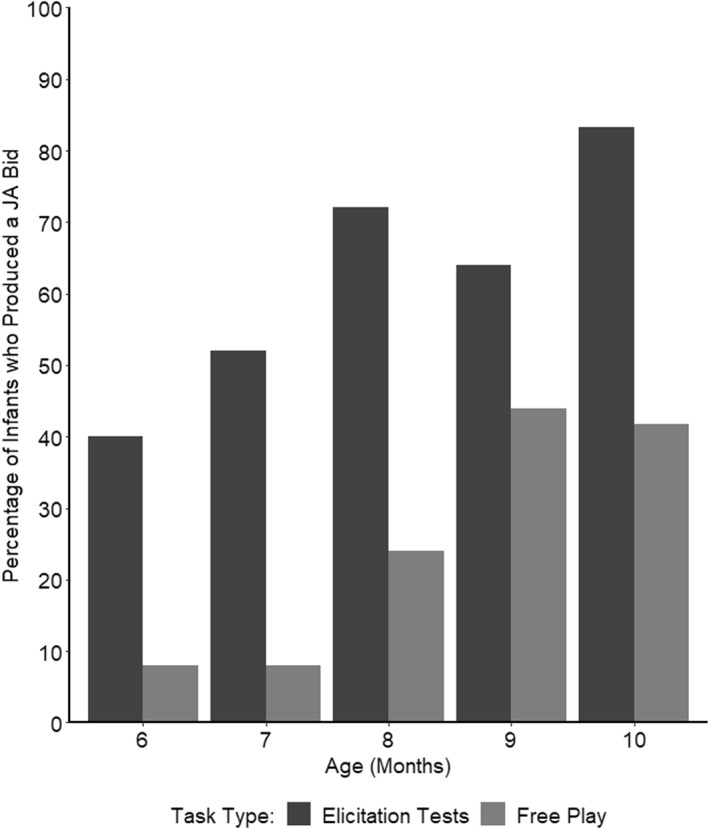
Percentage of Infants who Produced a Joint Attention Bid at each Age, Comparing Elicitation Tests (Collapsed) and Free Play. Elicitation test scores consist of the highest score received by each infant across the three elicitation tests combined for each age.

Next, we explored whether there were any differences in the number of infants who produced joint attention bids in the elicitation tests compared to the free play. Each infant only produced a maximum of one JA bid in any given elicitation test. In the free play, infants produced just one bid in the majority of cases in which a bid was identified (77.42%; see SM5.1 for further details). Figure 3 shows the percentage of infants who produced a joint attention bid at each age in the three elicitation tests (combined) and the free play.

A GLMM was specified, with the dependent variable being a binomial outcome, Joint Attention Bid Production, at each age. Task Type (a categorical variable: elicitation test or free play) and Age (continuous, centered) were included as fixed effects, and Participant was included as a random effect. A Tukey's HSD post hoc test examining differences between task types found that infants were significantly more likely to produce joint attention bids in the elicitation tests compared to in free play (log odds = 1.83[1.21,2.44], *p* < 0.001).

To investigate whether there were differences between task types at each age of assessment, a follow‐up GLMM was specified with an interaction between Task Type and Age (here, a categorical variable) included as a fixed effect, and participant as a random effect. Tukey's HSD post hoc tests examining differences between task types at each age found that the number of infants who produced joint attention bids was significantly lower in the free play than in the elicitation tests at all months except 9 months (*p* = 0.15), and in all cases the lower bound of the 95% confidence intervals for the effect sizes were above the threshold of at least a small effect size (6 months: log odds = 2.07[0.41,3.74], *p* = 0.015, 7 months: log odds = 2.57[0.91,4.23], *p* = 0.003, 8 months: log odds = 2.15[0.86,3.44], *p* = 0.001, 10 months: log odds = 1.99[0.62,3.36], *p* = 0.004).

Overall, the results showed that infants were significantly more likely to produce joint attention bids in the elicitation tests compared to the free play, despite the free play period lasting approximately twice as long as the elicitation tests combined. This was the case at every age except 9 months.

### Comparing Individual Elicitation Test Scores and Free Play Scores

**FIGURE 4:**
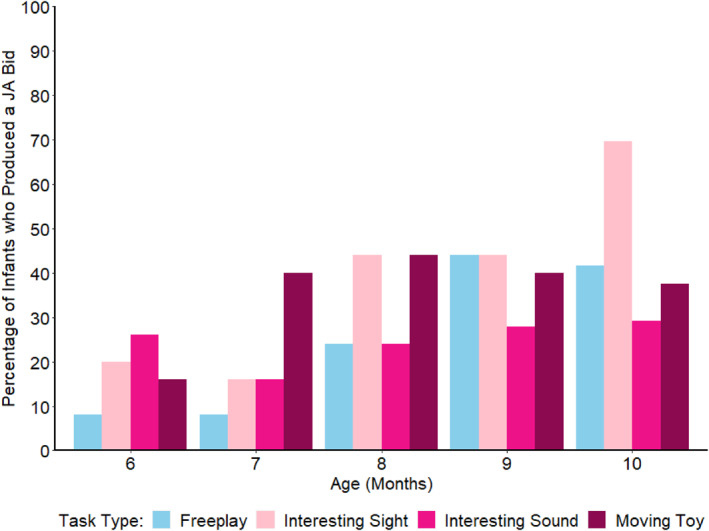
Percentage of Infants at each Age who Produced a Joint Attention Bid for each Elicitation Test and Free Play at each Age.

We next compared each individual elicitation test and free play. Figure 4 displays the percentage of infants at each age who produced at least one joint attention bid in each of the three elicitation tests separately and the free play.

A GLMM was specified, with the dependent variable being Joint Attention Bid Production. Task Type (a categorical variable with each of the three elicitation tests and free play as distinct categories) was included as a fixed effect, Age (centered) as a continuous fixed effect, and Participant as a random effect. Tukey's HSD post hoc tests on Task Type revealed no significant differences in Joint Attention Bid Production across the three elicitation tests and the free play.

### Examining the Consistency of Joint Attention Bid Production as Infants Aged

**FIGURE 5:**
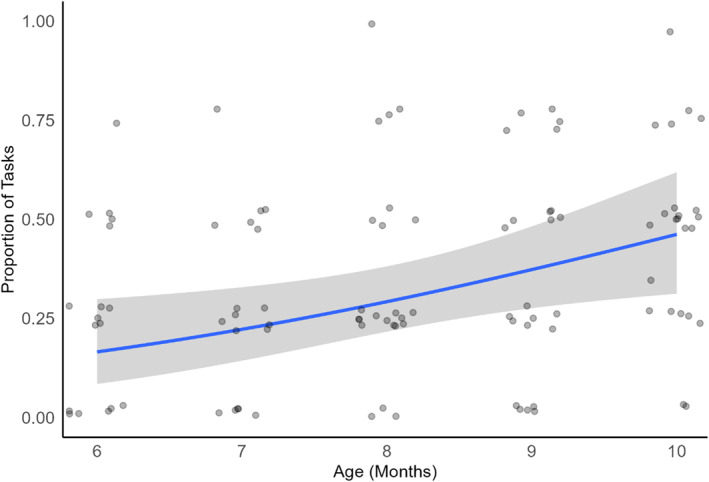
Proportion of Tasks in which Infants Produced a Joint Attention Bid at each Age. Dots represent actual data points (jittered for visibility). The blue line and gray area represent the estimated values and confidence intervals of the model, respectively.

Next, we examined whether there was an increase with age in how consistently infants produced joint attention bids. The number of tasks in which infants produced a joint attention bid on a scale from 0 to 4, including each elicitation test and free play, was used as an index of consistency. Figure 5 displays the proportion of tasks in which infants produced a joint attention bid over the period of assessment.

To investigate whether there was a change with age in the proportion of tasks in which infants produced a joint attention bid, two GLMMs were specified. The outcome variable was the proportion of tasks in which infants produced a joint attention bid, with an offset term to account for the few cases in which participants did not complete all tasks. In the first model, Age (centered) was a continuous fixed effect and Participant was a random effect. Age (centered) was a significant predictor of Joint Attention Bid Production in this model (log odds_baseline_ = −2.28[‐2.55,‐2.03], *p* < 0.001; log odds_age_ = 0.01[0.01,0.02], *p* < 0.001), with joint attention bids being produced in an increasingly large proportion of the tasks as children aged. Next, we examined whether there were any rapid developments with age in the proportion of joint attention bids across tasks, as indexed by a significant change between consecutive months. In a second GLMM, Age was fit as a categorical fixed effect and Participant was a random effect. Tukey's HSD post hoc tests on Age revealed significant increases in infants' production of joint attention bids only between non‐consecutive months (between 6 and 9, 6 and 10, 7 and 9, and 7 and 10 months), The lower bound of the 95% confidence intervals for the effect size was above the threshold of a small effect size (log odds<0.36) only for the comparison between 6 and 10 months. There was no significant difference between any two consecutive months. For models with data from the elicitation tests (collapsed) and free play separately, see SM5.

Thus, the consistency with which infants produced a joint attention bid, as indexed by the proportion of tasks in which a joint attention bid was produced, increased with age, with no sudden developments between consecutive months.

### The Production of Non‐communicative Looks

**FIGURE 6:**
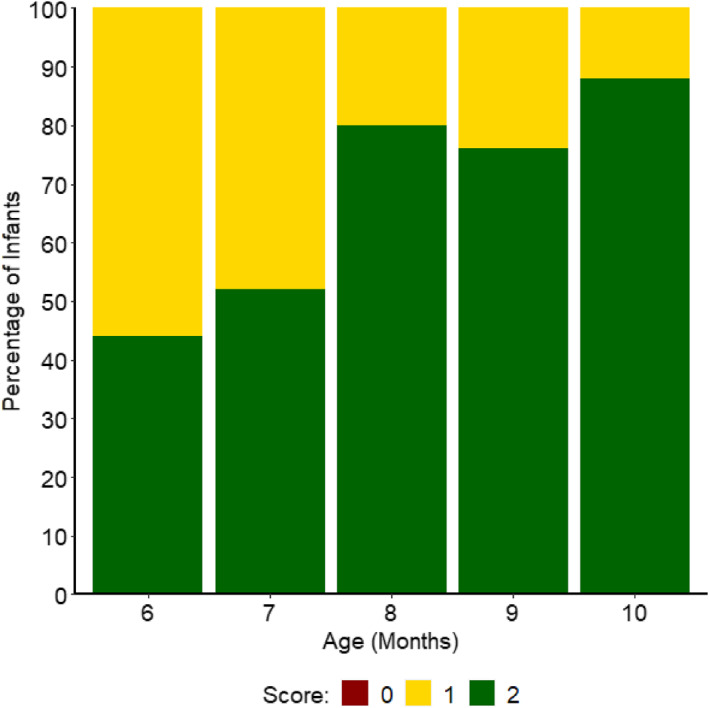
Percentage of Infants who Received a Score of “0”, “1” or “2” as their Highest Score for Joint Attention Bids at each Age, Collapsed across Tasks.

Lastly, we explored infants' production of non‐communicative looks to the adults about the stimuli (i.e., scores of “1”). Figure 6 shows the highest score across all four tasks collapsed received by each infant at each age (for individual infants' scores at each month, and for the results for non‐communicative vs. communicative looks for each task individually, see SM5.1).

All infants received a score of at least “1” at each age. Thus, for those who would relax the requirement of communication as a necessity for a joint attention bid, 100% of infants had produced a joint attention bid by 6 months.

## Discussion

Communication has been proposed as the means by which infants (and adults) establish the jointness in joint attention (Carpenter and Liebal 2011; Eilan, n.d.; see also Siposova and Carpenter 2019). We thus used a conservative definition of joint attention involving communication to investigate when young infants first begin to make bids to initiate joint attention with their partner. In so doing, we investigate the very beginnings of both joint attention and referential communication in development. This study challenges the notion that infants become capable of initiating joint attention only at 9 months of age (Hubley and Trevarthen 1979; Tomasello 1999). It also highlights how these looks are infants' earliest means of referential communication (Carpenter and Liebal 2011; Jones and Hong 2001), but at an earlier age than in previous findings. Not only had almost all infants (92%) made a joint attention bid before 9 months, but a substantial majority (72%) had made a joint attention bid by 7 months, and close to half (44%) had made a joint attention bid by 6 months of age. In further contrast with the notion of a “9‐month revolution” (Tomasello 1999), there was no sudden increase in infants producing joint attention bids between any two consecutive months.

These findings complement previous research that argues for a gradual emergence of joint attention starting before 9 months (de Barbaro et al. 2013; de Barbaro et al. 2016; Rossmanith et al. 2014; Striano and Bertin 2005). This study also extends that research in the following ways. It used a conservative definition of joint attention requiring active, referential communication, and a method in the elicitation tests that removed adult scaffolding. It ruled out looks that were only in response to something the adult had done, and it provided evidence of joint attention bids in a larger proportion of infants earlier than has been shown previously, and to different types of stimuli, including both visual and auditory stimuli. This study thus provides evidence that the age of emergence of both joint attention and referential communication may be earlier than previously identified.

With age, infants produced joint attention bids across an increasing number of tasks. This suggests that infants' capacity to produce joint attention bids becomes more consistent during this period, or that infants are increasingly motivated to engage communicatively with others, or both. Still, while over half of infants (56%) produced joint attention bids at every session after producing their first joint attention bid, not every infant who produced a joint attention bid during an early session then went on to do so at every subsequent session, and relatively few infants even at the older ages produced joint attention bids in all four tasks, though this variation may be due to factors such as mood and interest in the stimuli. The idea of a joint attention “revolution” (Tomasello 1999) occurring at around 9–12 months could be reinterpreted as being more about consistency and the emergence of a number of other, additional joint attention behaviors (like shows and points), rather than the initial emergence of the first instances of joint attention (see Salter and Carpenter 2022, for further discussion about the emergence of early referential gestures in these same infants).

Taken together, these findings contribute to several active theoretical debates about joint attention and communication. First, previous conceptual work has stressed that for joint attention to be truly *joint*, it requires active sharing through communication (Carpenter and Liebal 2011; Eilan, n.d.; see also Siposova and Carpenter 2019). Here we have shown that this is well within both the capability and the motivation of even very young infants. The communicative facial expressions and vocalizations that accompanied infants' looks provide evidence of active sharing—an attempt to initiate “truly joint” joint attention, going beyond just alternation of gaze alone, which could just involve alternation of attention or “checking” looks (Carpenter and Liebal 2011; Gabouer and Bortfeld 2021; Tomasello 1995).

Second, the finding that these communicative acts emerge months earlier in development than other early communicative behaviors such as showing and pointing contributes to debates about what cognitive mechanisms are involved in early communication. For example, previous work has linked the onset of intentional communication around 9 months with means‐ends understanding, which emerges around the same age when assessed through tasks involving obtaining a distal object by pulling on a cloth under the object or a string attached to it (E. Bates et al. 1979; Jones and Hong 2001). However, the earlier age of emergence of communicative, referential looks in this study suggests that we either need to re‐consider whether means‐ends understanding is a pre‐requisite for communication, or consider whether infants may have a more minimal level of means‐ends understanding prior to passing these specific means‐ends tasks. Regardless, the very early production of joint attention bids suggests that theories of communication that require complex cognitive inferences like recursive mindreading (e.g., Tomasello 2010) will struggle to explain our results. Rather, as a number of theorists have suggested (e.g., Bar‐On 2013; Breheny 2006; Moore 2017), a more minimal account of communication may be necessary, and may be better able to account for these findings.

The current findings also relate to a further theoretical debate: the “message” behind (or function of) early joint attention looks and gestures (e.g., Begus and Southgate 2012; Graham et al. 2021; J. A. Hobson and Hobson 2007). Conceivably, the looks might have communicated a variety of messages about the stimulus, including declarative (e.g., “That's cool!”), informative (“Look at that!”), interrogative (“What is that?”, “Should I be afraid?”), or imperative (“I want it!”) messages. The finding that the only facial expression observed to accompany joint attention bids was smiles, rather than other (e.g., quizzical or insistent) expressions, might suggest that they served a declarative function. However, it is important to be cautious with attempts to gloss the looks, since these looks are not necessarily univocal signals that are straightforwardly interpretable as propositions.

Incidentally, the finding that the only accompanying facial expressions were smiles additionally counters a possible alternative explanation for these looks: that they were non‐communicative social referencing looks rather than communicative joint attention looks. The looks were produced with an expression of the infants' already‐existing attitude—an anticipatory smile—rather than, as in the case of social referencing, seeking to assess another's attitude. Social referencing is still a viable option for the function of the spontaneous non‐communicative looks to the adults about the objects (those indexed by a score of “1” in this study). Those looks could have served as social referencing looks (to assess the other's attitude about the objects), as checking looks (to confirm the other's presence and/or attention), or as non‐communicative joint attention bids (see Siposova and Carpenter's, 2019, “mutual attention” level of joint attention).

Methodologically, the study provided a conservative definition of joint attention bids and a coding scheme rooted in objective, publicly observable behaviors, that required coordinated, communicative, referential facial expressions and/or vocalizations along with the looks. The study was also original in its use of novel tests to elicit joint attention bids. We found that significantly more infants produced joint attention bids, at earlier ages, in the elicitation tests than in the free play, with a large effect size. Thus, coding joint attention from free play, as most researchers do, may drastically underestimate infants' true capabilities. There are several plausible reasons for these differences. First, free play involves a complex situation, typically with multiple objects present (Koşkulu et al. 2021), as well as interaction partners (typically caregivers) who respond dynamically, and who may intervene if perceiving little or no social initiation on the part of the infant (Bruner 1977; Vygotsky 1978). Thus, infants' true capabilities are masked, or are very difficult to clearly identify. In contrast, the elicitation tests strip back much of the environmental complexity and adult scaffolding. Infants encountered a single salient stimulus, and an interaction partner who allowed them an extended period (around 30 s) in which to produce a response, providing more optimal conditions for examining infants' own spontaneous capabilities. Second, in free play, infants are permitted to interact manually and orally with the objects, and may struggle to disengage their visual attention in order to look to their caregiver (de Barbaro et al. 2016). In contrast, in the elicitation tests, the stimuli are out of infants' reach, and the stimuli regularly deactivated, providing infants with an opportunity to disengage their attention and look communicatively with minimal cognitive effort. A final reason that free play interactions may not regularly elicit many joint attention bids is because, as demonstrated by Yu and Smith (2013; see also 2017), engagements between infants and caregivers involving objects can typically proceed with minimal eye contact, with visual attention to each other's manual activity sufficing for an interaction to continue. In contrast, the elicitation tests always involved a stimulus that was novel to the infant and the interaction, which the adult was not already looking at, with no opportunity for manual exploration or adult activity to watch, thus creating a situation in which a joint attention bid was a more likely response. Despite these advantages, it is also possible that the unusual nature of the situation in the elicitation tests (i.e., an interaction partner appearing not to notice a salient stimulus) may have slightly offset these benefits, and thus that even the elicitation tests might represent an underestimation of infants' true abilities. It is important to note that in discussing the advantages of the joint attention elicitation tests, our aim is not to diminish the importance of naturalistic, observational data, but rather to highlight how experimental methods might reveal new insights that are difficult to glean from free play. Testing infants in both contexts is informative.

The novel methods and findings of the study open new directions for future research. First, a natural and needed next step is to use these paradigms with even younger infants. Given that all infants in this study had produced at least a non‐communicative look, if not a joint attention bid, by 6 months, it is plausible that the elicitation tests would elicit these looks in younger infants too, but it is not clear quite how young. Conducting this paradigm with younger infants will enable us to establish when in development infants first start to produce joint attention bids, and whether this does occur prior to 6 months of age for some infants. It will also allow investigation of whether joint attention bids are reliably preceded by the ability and motivation to produce non‐communicative looks. Previous work has shown that some infants as young as 5 months (approximately 30%) can produce non‐communicative looks in a free play context (Striano and Bertin 2005), but, for the reasons noted above, the new paradigm that has been developed here may be even more effective at eliciting looks than free play. Because all infants already produced non‐communicative looks at their first visit (and because many infants also produced communicative joint attention bids at the first visit), we could not examine their relation to the onset of communicative joint attention bid production.

A further important feature of this study was the use of varied stimuli across different tasks, including sight, sound, and motion. An issue for future exploration is how infants come to be capable of sharing different kinds of stimuli. Recent theoretical accounts have drawn attention to the multi‐modal nature of joint attention (Battich et al. 2020; Botero 2016; Gabouer and Bortfeld 2021; Siposova and Carpenter 2019), but there remains little research into infant responses to non‐visual stimuli such as sounds, smells, or tactile sensations. Infants' lives are replete with shared experiences of varied stimuli, with different stimuli and modalities often experienced simultaneously. Thus, our understanding of the development of joint attention would benefit from seeking to understand how infants communicatively share sounds, tastes, and smells, if they do. Furthermore, there is variety in how different stimuli are experienced. Consider a joint attention situation involving a sound being emitted from a specific location, versus a non‐localized sound, such as the sound of falling rain, or of a crowd. There is little work that considers how infants (or indeed older children or adults) might share different kinds of sensory experiences. This may be because of the practical challenges of working with different stimuli, both in terms of the implementation of experimental protocols and the development of behavioral coding schemes. For example, our experience of coding responses to sounds was that it was more complex than coding visual stimuli due to the lack of a clear “anchoring point” in space as the target of the look. Meeting this challenge will thus require creative ways of approaching experimental design and behavioral coding in order to assess infants' responses to varied stimuli, as well as investigation of infants' responses to different stimuli in their daily lives.

There are also further questions to be explored regarding the factors that influence infants' joint attention bid production. Here we have demonstrated a difference between free play and elicitation tests, but further specific task features and their effects could be isolated. These include the type and timing of responses from interaction partners (in both naturalistic and controlled situations), the spatial layout of the situation, individual differences in how much time infants spend engaging with objects, and the aforementioned influence of different stimulus types and modalities. The elicitation tests could also be supplemented with eye‐tracking to obtain a more precise measure of gaze timing and target of infants' gaze during the procedure.

Finally, it is important to note that the sample used is from a “WEIRD” population (Henrich et al. 2010; Nielsen et al. 2017); all infants were living in a rich, Western, democratic nation, with educated parents. It is possible that the observed developmental trajectory of joint attention bids may be a particular feature of this cultural context. It has been suggested that interactions in non‐WEIRD cultures rely less on the visual modality, with a greater emphasis on physical contact (Akhtar and Gernsbacher 2008; Botero 2016; Little et al. 2016), which may lead to differences in the production of joint attention bids in different contexts. However, studies have also found that, across different cultures, infants at around 1 year of age engage in joint attention behaviors such as communicative pointing (Callaghan et al. 2011; Liszkowski et al. 2012), which may suggest minimal variability due to cultural context, at least in the visual situations in which they were tested. Further work could attempt to use this paradigm (adapted as needed) in different cultural contexts to examine whether there are different patterns of emergence.

To summarize, this study builds on recent theoretical work on the role of communication in joint attention to develop a new approach to investigating infants' ability to make bids for joint attention using communicative looks. These looks do not just involve gaze alternation between an agent and an object, but rather may constitute what are among infants' earliest communicative “comments” about the world to others, which serve to establish an aspect of the world as shared. The study has provided novel, effective methodological tools for eliciting joint attention bids, and presents evidence that some infants are capable of producing communicative joint attention bids to try to initiate joint attention by at least 6 months of age, and that infants' capacity and/or motivation to engage in joint attention emerges gradually over the course of their first year. These findings represent an important step toward developing a deeper understanding of a vital period of human social development, one which has immense significance for our understanding of the origins of human communication, sociality, and culture.

## Author Contributions

**Gideon Salter:** conceptualization, data curation, formal analysis, funding acquisition, investigation, methodology, project administration, resources, software, visualization, writing – original draft. **Malinda Carpenter:** conceptualization, investigation, methodology, resources, supervision, validation, writing – review and editing.

## Ethics Statement

This research received ethical approval from the School of Psychology and Neuroscience Ethics Committee and the University Teaching and Research Ethics Committee at the University of St Andrews (reference code: PS13951).

## Conflicts of Interest

The authors declare no conflicts of interest.

## Supporting information

Supporting Information S1

Supporting Information S2

Supporting Information S3

Supporting Information S4

Supporting Information S5

## Data Availability

Data are made available as part of the supplementary materials.
