# Supplementary material for: The Developmental Origins of Joint Attention: Infants' Early Joint Attention Bids
Source: Infancy. 2025 Mar 26;30(2):e70012. doi: 10.1111/infa.70012 (PMC11947298; doi:10.1111/infa.70012)
Supplement: Supplementary file 1 — Supporting Information S1 [file INFA-30-0-s003.docx]

**Supplementary Materials 1**

Materials and testing room setup for the Free Play and Joint Attention Elicitation Tests. A 15cm ruler is presented with each object for scale.

**Figure S1.1**

*Example Toys from Free Play*


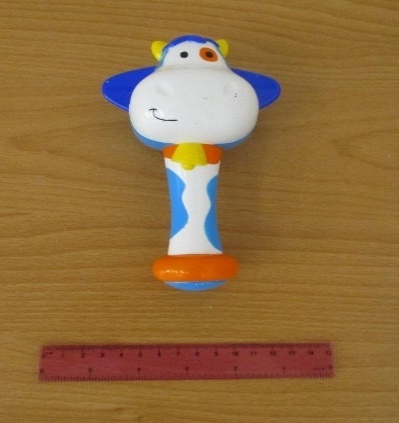

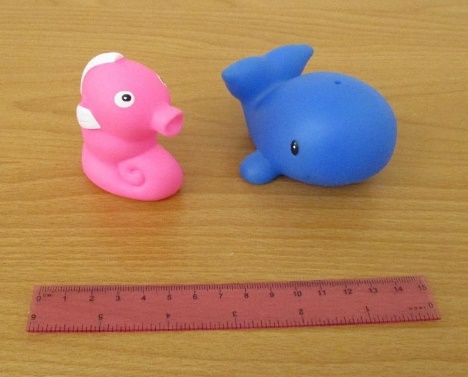

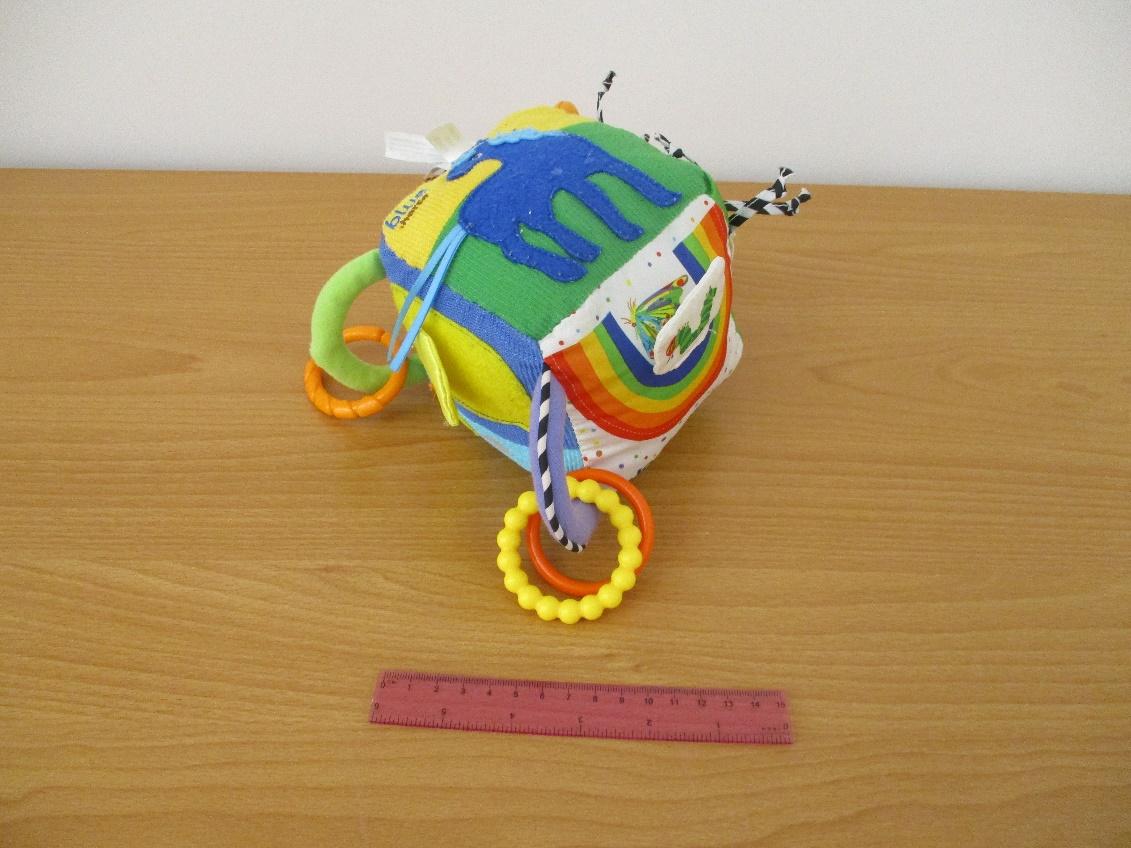

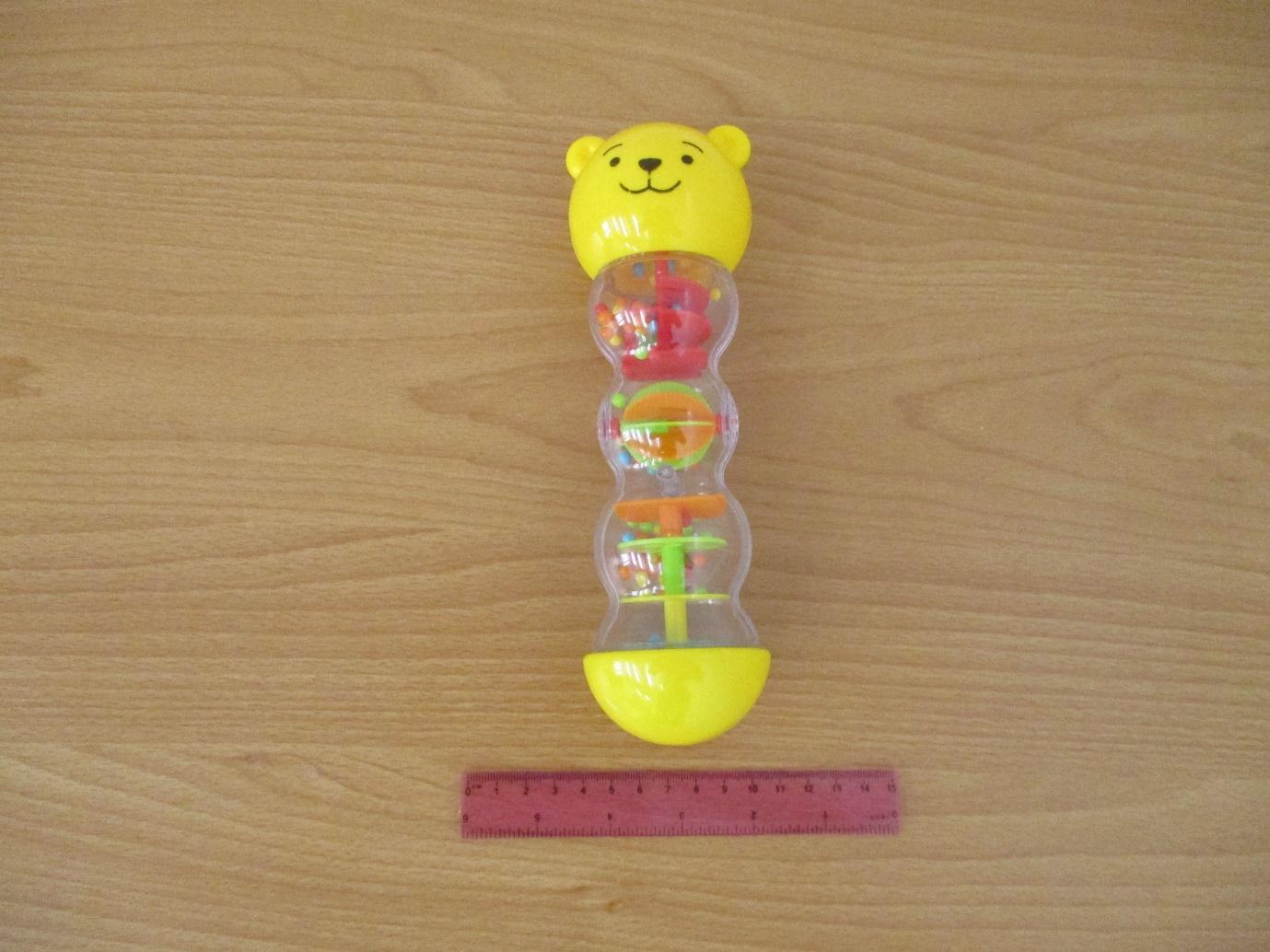

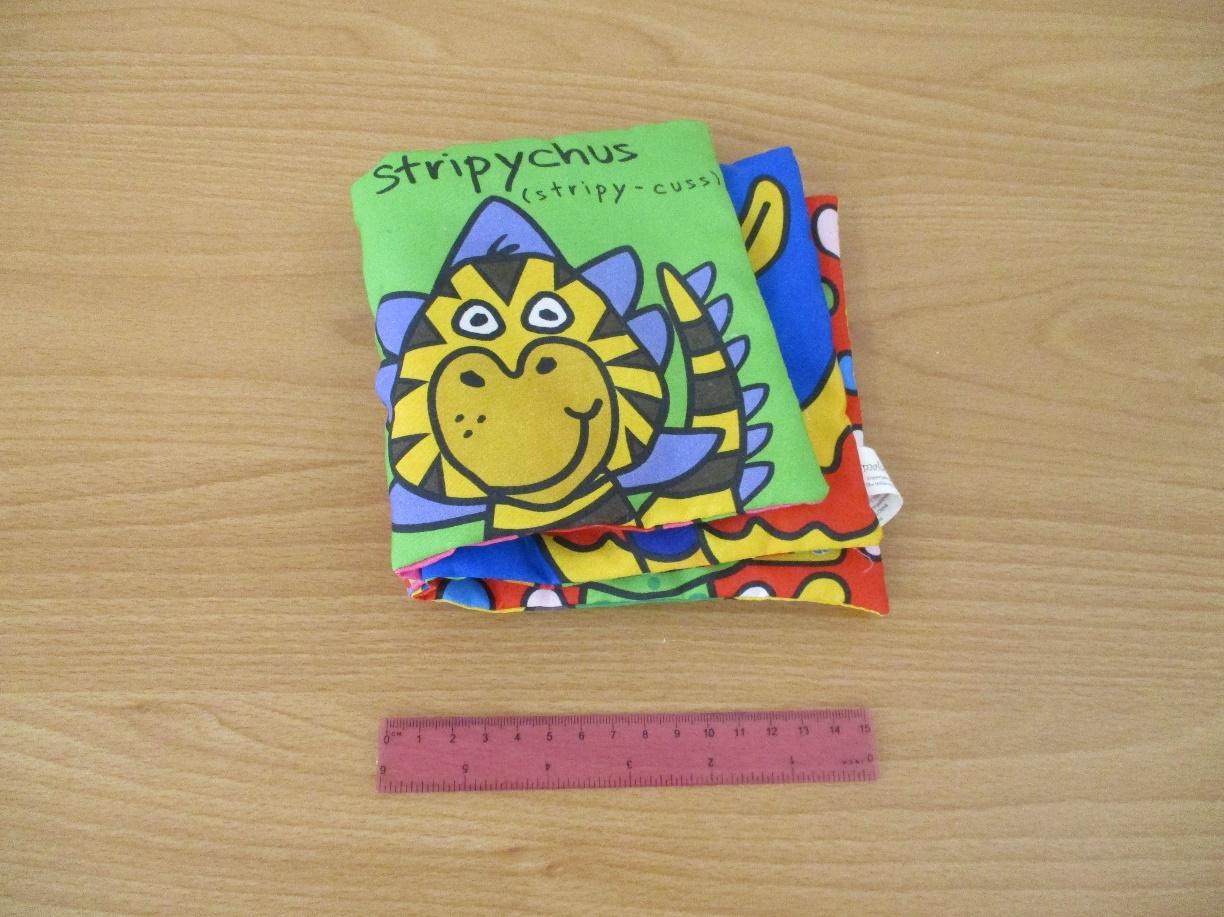


**Figure S1.2**

*Light box used in the Interesting Sight Test*


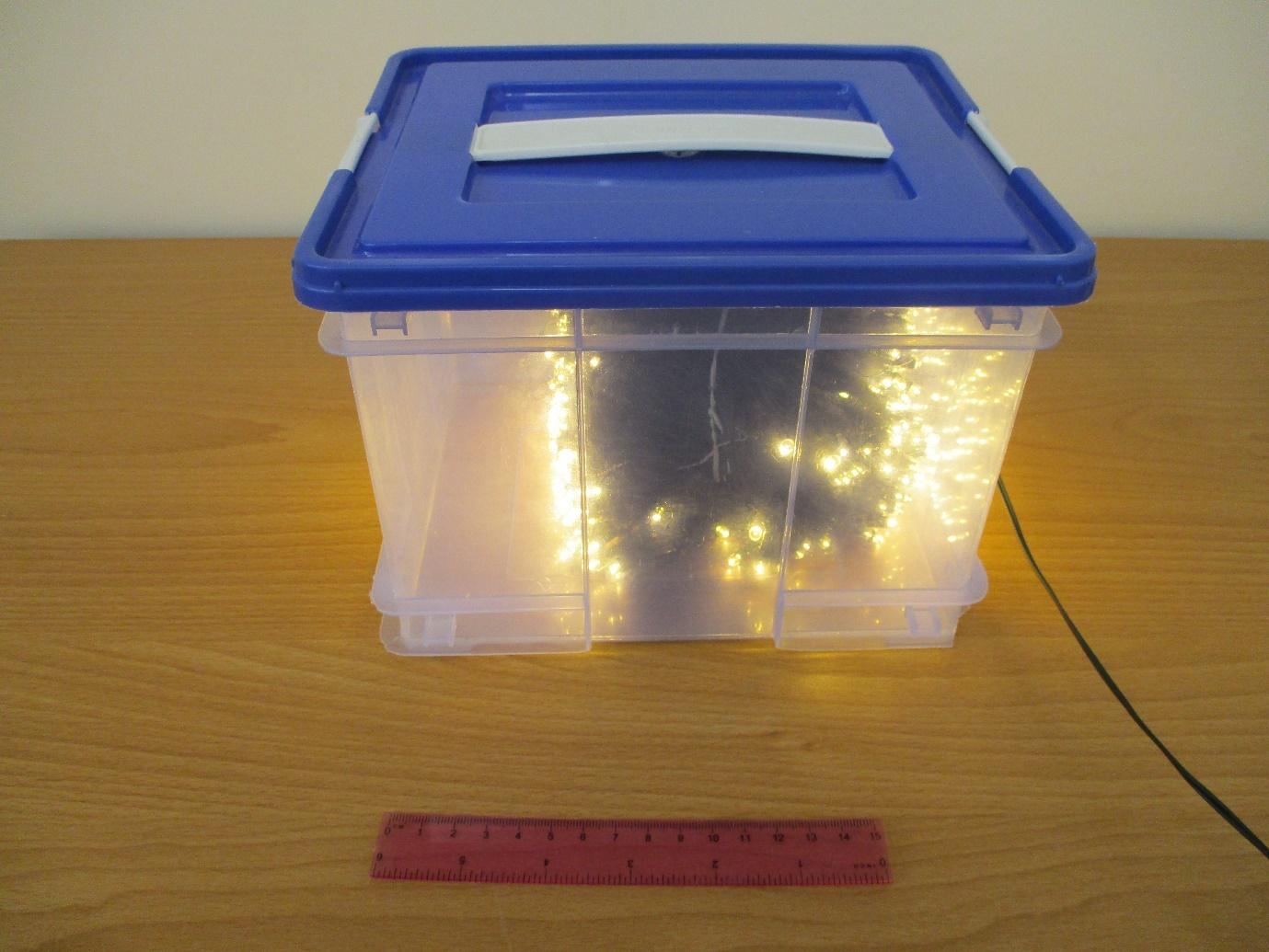


**Figure S1.3**


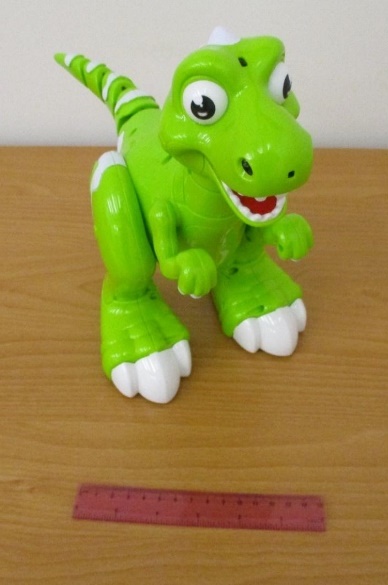
*Remote-controlled toys used in the Moving Toy task*


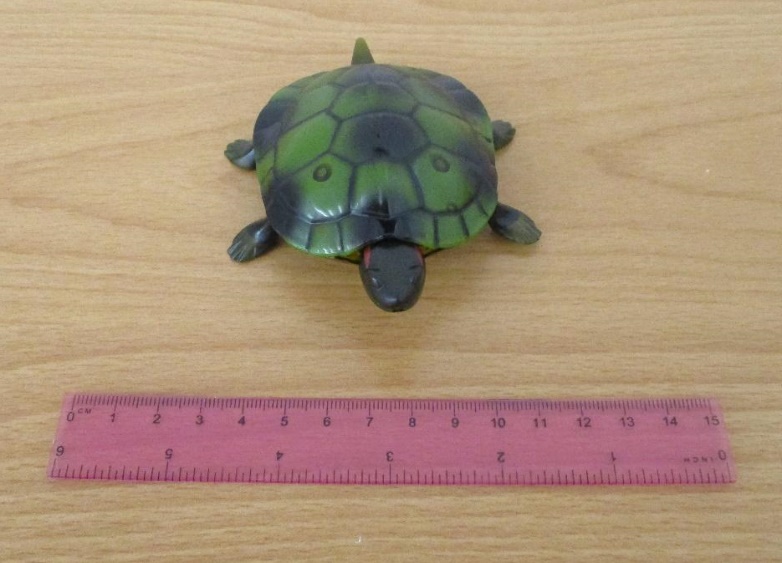


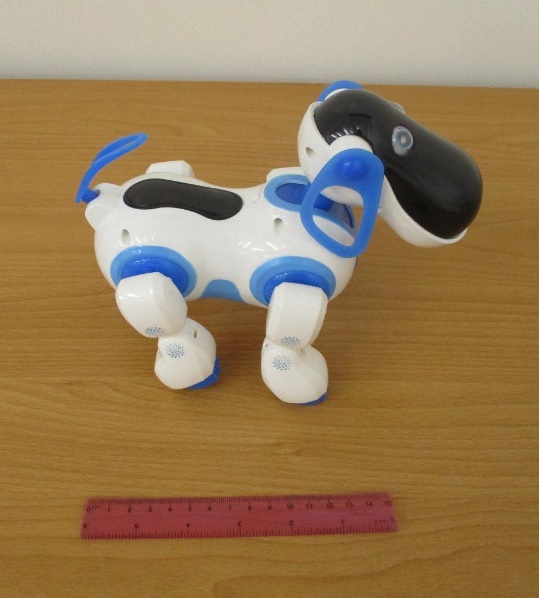


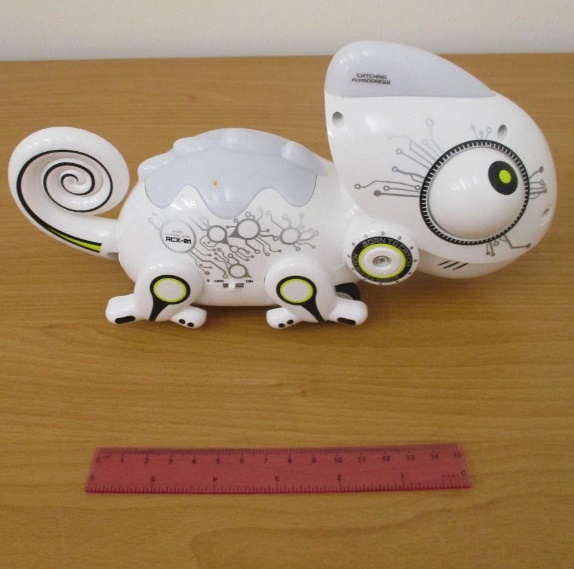


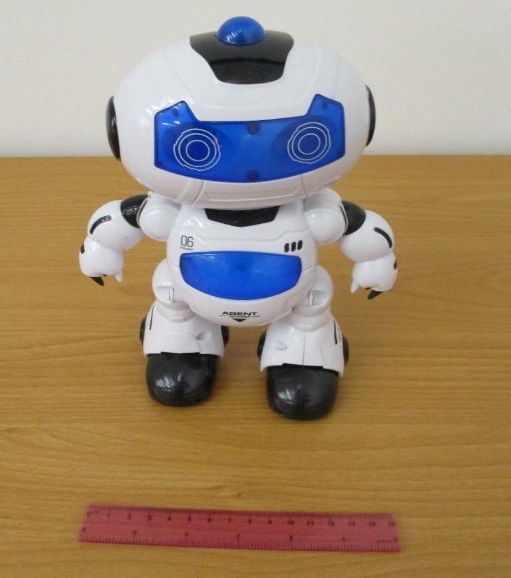


**Figure S1.4**


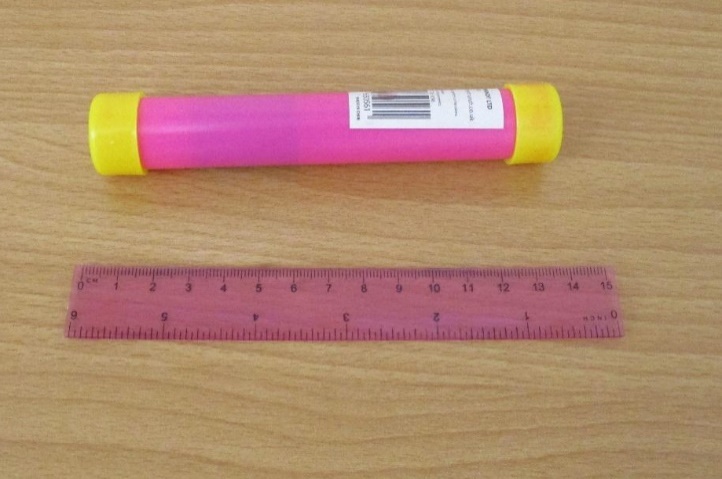
*Stimuli used in the Interesting Sound Test*


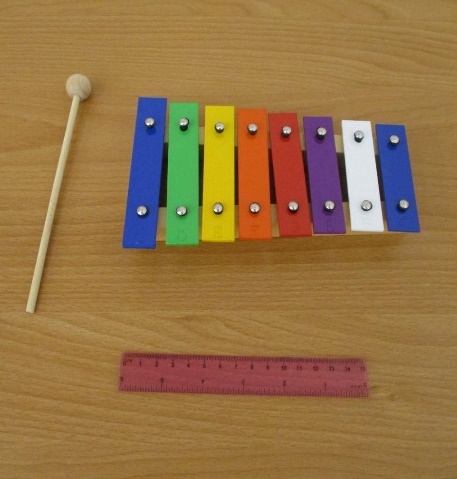


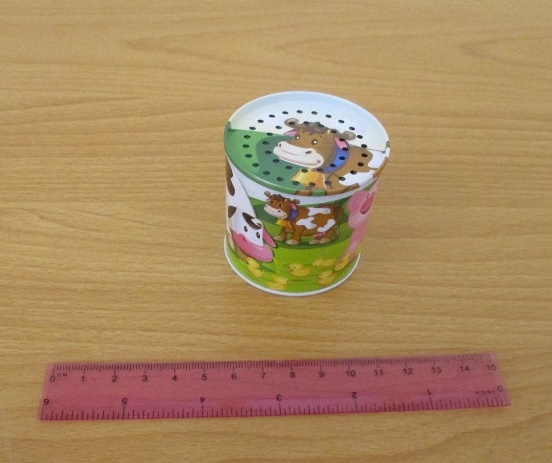


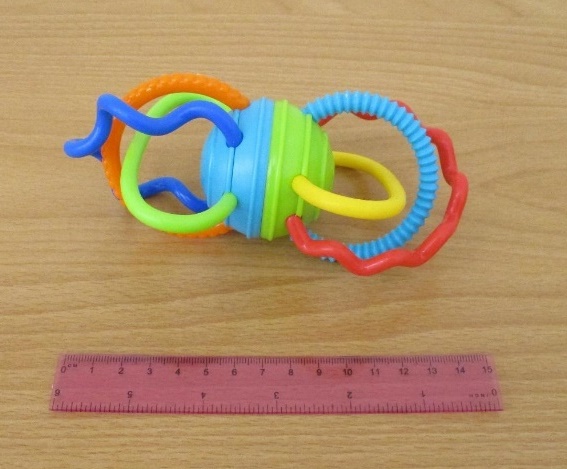


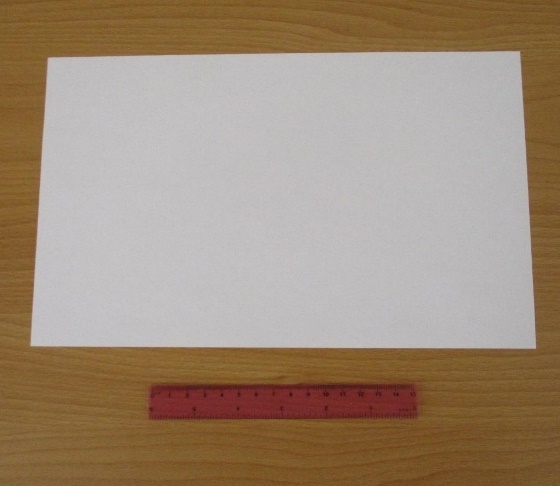


*Note.* In order, the objects are: a “Groan tube” that produces a groaning sound when inverted, a xylophone that produces musical notes when struck, a “Moo tube” that produces a mooing sound when inverted, a toy that clicks loudly and repeatedly when twisted, and an A4 sheet of paper that was crumpled repeatedly to produce a noise.

**Figure S1.5**

*Testing Room and Setup for Free Play*


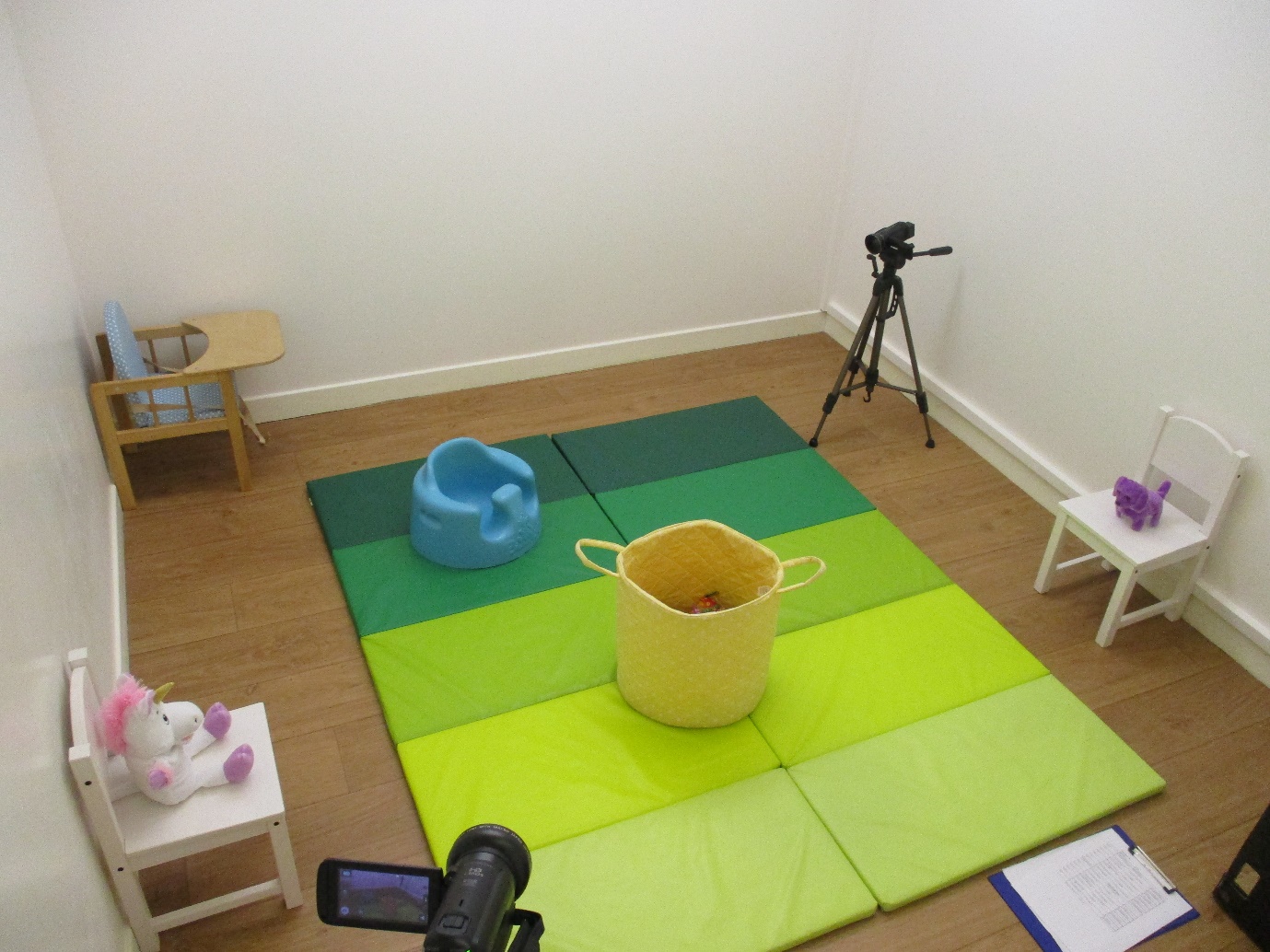


**
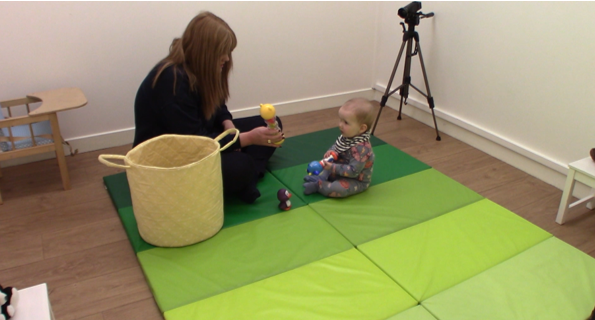
**

**Figure S1.6**

*Testing Room and Setup for Interesting Sight and Moving Toy Tests*

*
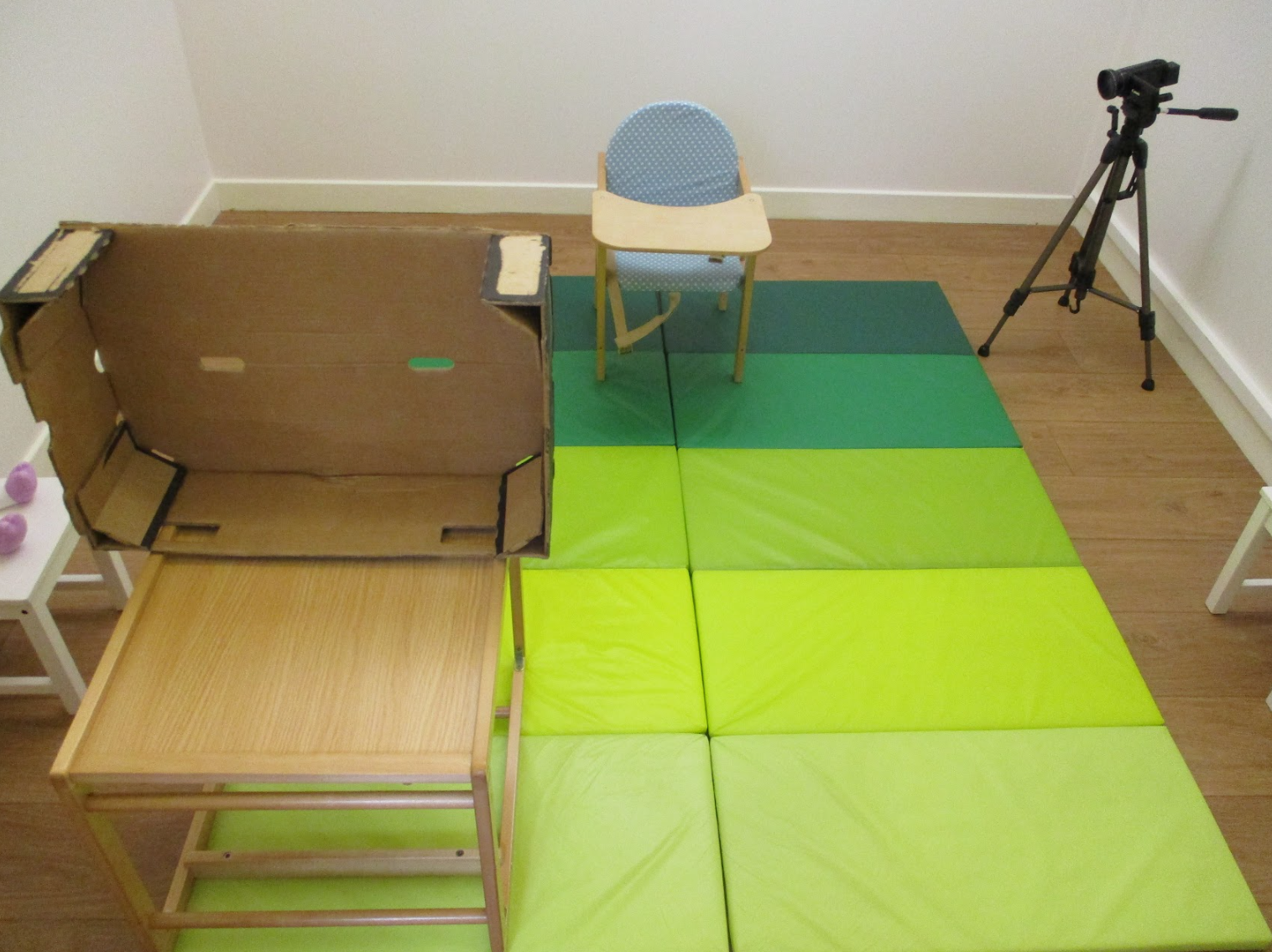
*


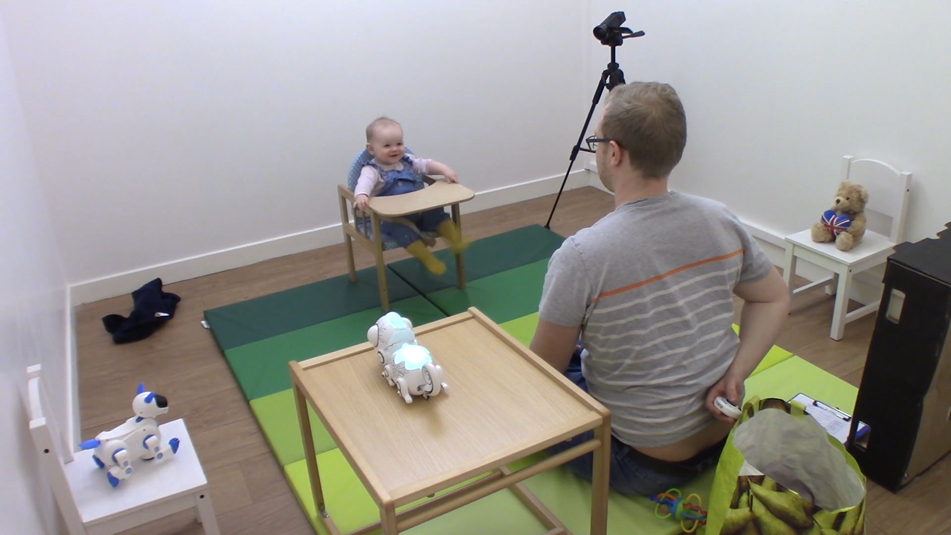


**Supplementary Materials 2**

**Behavioural coding scheme for Joint Attention Bids**

**Figure S2**

*Flow Chart for the Coding of Joint Attention Bids*

*
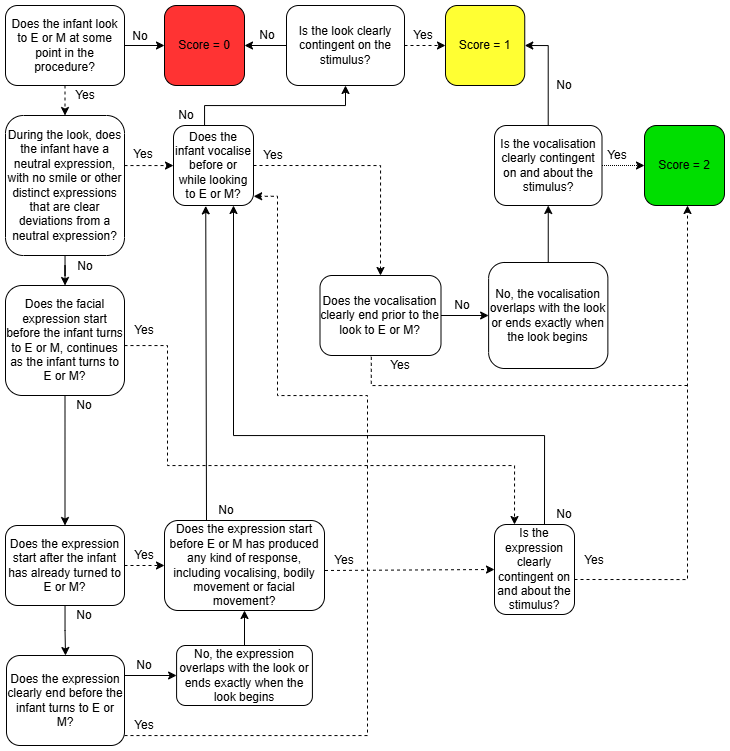
*

*Note.* “E” refers to experimenter, and “M” refers to mother.

Further points of guidance.

- ***Is the look to E or M clearly about* *the toy/stimulus?*** The look needs to occur as the first look after the infant has attended to the toy/stimulus. It needs to be clear that the look was about the toy/stimulus and not some other object. The length of time before the look to E/M does not matter for the visual stimuli: it is acceptable for the infant to look continually at the stimulus for any length of time and then look to E/M.
- ***Previously performed behaviours.*** Look-accompanying behaviours to E/M, whether facial expressions or vocalisations, are sometimes present prior to the onset of the stimulus. If so, consider whether the behaviours that accompany the look are clearly distinct communicative acts about the stimulus, or are just continuations of the same behaviour.
- ***Resetting between phases of the tests:*** Each test involves the stimulus being activated three times (light box on, moving toy activated, sound made). Each activation “resets” the coding scheme (i.e., previous responses from E during prior activations do not delegitimise infants’ subsequent responses). Resets can also occur if infants have looked to E, reacted, look back to the stimulus, and look back to E again. The main stipulation is that they are clearly reacting *about the stimulus*.
- ***Looks to the adult’s face.***As long as the looks are clearly identifiable as being to E’s or M’s face, they count. It does not matter if they are brief, as long as they involve at least a short fixation on the adult’s face.
- ***Coding the Interesting Sound task.*** One of the tests, Interesting Sound, is unique, in that there is no visible object that serves as a visual target of the joint attention look. Thus, the coders need to establish that the look to E/M is in response to the sound and not some other potential stimulus in the room. One piece of evidence to use is whether the look occurs promptly after the sound is produced and contingently on it. We opted not to provide an arbitrary time threshold within which the look had to occur after an activation of the sound, instead leaving this to the judgement of the coder. This was because latency is not a fool-proof indicator of whether the look is about the stimulus; for example, infants sometimes paused, listening, in response to the sound before looking to E/M, or searched for the source of the sound before looking to E/M.
- ***Coding from free play.*** In free play, particular care needs to be paid to whether the look to the mother’s face is in response to the activity of the mother. If you cannot be certain whether it is or is not in response to the activity of the mother, code conservatively and score a “0”. The target of the initial look to a stimulus (e.g., a toy or the noise made by a toy) must be clearly identifiable. If it is not clear, code conservatively and score a “0”.

***SM2.1 Coding of looks back to stimulus***

- JA bid followed by a look back to the stimulus.
  - This behaviour is counted if the first gaze shift after the look to the social partner (experimenter or mother) is the stimulus from which the first look occurred.
  - A gaze shift is a single saccade or continuous movement of the eyes until fixation on a particular location in space.
  - This includes cases in which the infant looks back when the stimulus reactivates.
  - If the infant’s first fixation after the look to the social partner is on anything other than the target stimulus then this cannot be counted, even if they end up looking back at the stimulus.
  - This behaviour is coded with a score of “1”.
- JA bid that is not followed by a look to the stimulus.
  - The first gaze shift is not back to the original stimulus.
  - This behaviour is coded with a score of “0”.

**Supplementary Materials 3**

**Inter-rater Reliability**

**Table S3**

*Reliability for All Tasks Together, and for the Elicitation Tests and Free Play Separately*

| **Task Type** | **Cohen’s *ĸ*** |
| --- | --- |
| All Tasks | 0.71 |
| Elicitation Tests | 0.72 |
| Free Play | 0.56 |
| Looks Back to Stimulus | 0.86 |

The first author was the main coder and the same naïve undergraduate second coder independently coded all tasks to assess inter-rater reliability. For the elicitation tests, 100% of tests were coded for reliability; for the free play, 33% of the videos were coded for reliability. For the coding of looks back, the first author was the main coder, and a different naïve undergraduate second coder coded 20% of cases. The main coder’s coding was used in all cases.

**Supplementary Materials 4**

**Tables of Model Coefficients**

**Table S4.1**

*Output of GLMM Modelling Production of Joint Attention Bids at each Age Across all Three Elicitation Tests and Free Play Combined, Age as Continuous Predictor*

|  | Joint Attention Bid Score | | | |
| --- | --- | --- | --- | --- |
| Predictors | Odds Ratios | SE | CI | *p* |
| (Intercept) | 2.52 | 0.66 | 1.51-4.20 | <0.001*** |
| Age (days, centred) | 1.02 | 0.01 | 1.01-1.03 | <0.001*** |
| Observations | 124 | | | |
| Marginal R^2^ / Conditional R^2^ | 0.178 / 0.254 | | | |

*Note.* ****p* < 0.001.

**Table S4.2**

*Output of GLMM Modelling Production of Joint Attention Bids at each Age Across all Three Elicitation Tests and Free Play Combined, Age as Categorical Predictor*

|  | Joint Attention Bid Score | | | |
| --- | --- | --- | --- | --- |
| Predictors | Odds Ratios | SE | CI | *p* |
| (Intercept) | 0.77 | 0.34 | 0.32-1.83 | 0.55 |
| Age (7 months) | 1.43 | 0.85 | 0.44-4.61 | 0.55 |
| Age (8 months) | 5.91 | 4.07 | 1.53-22.77 | 0.010* |
| Age (9 months) | 4.61 | 3.05 | 1.26-16.84 | 0.021* |
| Age (10 months) | 10.41 | 8.12 | 2.26-47.98 | 0.003** |
| Observations | 124 | | | |
| Marginal R^2^ / Conditional R^2^ | 0.174 / 0.267 | | | |

*Note.* **p* < 0.05, ***p* < 0.01.

**Table S4.3**

*Output of GLMM Modelling Production of Joint Attention Bids, Comparing Elicitation Tests (Collapsed) and Free Play, Age as Continuous Predictor*

|  | Joint Attention Bid Score | | | |
| --- | --- | --- | --- | --- |
| Predictors | Odds Ratios | SE | CI | *p* |
| (Intercept) | 0.29 | 0.07 | 0.18-0.45 | <0.001*** |
| Age (days, centred) | 1.02 | 0.00 | 1.01-1.03 | <0.001*** |
| Task Type (Elicitation) | 6.21 | 1.95 | 3.35-11.50 | <0.001*** |
| Observations | 248 | | | |
| Marginal R^2^ / Conditional R^2^ | 0.300 / 0.313 | | | |

*Note.* ****p* < 0.001. Task Type was a categorical variable with two levels, “Free Play” (reference category) and “Elicitation.”

**Table S4.4**

*Output of GLMM Modelling Production of Joint Attention Bids, Comparing Elicitation Tests (Collapsed) and Free Play, Age as Categorical Predictor*

|  | Joint Attention Bid Score | | | |
| --- | --- | --- | --- | --- |
| Predictors | Odds Ratios | SE | CI | *p* |
| (Intercept) | 0.10 | 0.04 | 0.04-0.23 | <0.001*** |
| Age (7 months) | 1.43 | 0.70 | 0.55-3.73 | 0.466 |
| Age (8 months) | 3.63 | 1.75 | 1.41-9.34 | 0.007** |
| Age (9 months) | 4.89 | 2.38 | 1.89-12.68 | 0.001** |
| Age (10 months) | 7.46 | 3.73 | 2.80-19.89 | <0.001*** |
| Task Type (Elicitation) | 6.21 | 1.95 | 3.35-11.50 | <0.001*** |
| Observations | 248 | | | |
| Marginal R^2^ / Conditional R^2^ | 0.293 / 0.313 | | | |

*Note.* ***p* < 0.01, ****p* < 0.001. Task Type was a categorical variable with two levels, “Free Play” (reference category) and “Elicitation.”

**Table S4.5**

*Output of GLMM Modelling Production of Joint Attention Bids, Elicitation Tests versus Free Play, with an Interaction between Age and Task Type, Age as Continuous Predictor*

|  | Joint Attention Bid Score | | | |
| --- | --- | --- | --- | --- |
| Predictors | Odds Ratios | SE | CI | *p* |
| (Intercept) | 0.27 | 0.07 | 0.16-0.45 | <0.001*** |
| Age (days, centred) | 1.02 | 0.01 | 1.01-1.03 | <0.001*** |
| Task Type (Elicitation) | 6.43 | 2.07 | 3.42-12.08 | <0.001*** |
| Age (centred)* Task Type (Elicitation) | 0.99 | 0.01 | 0.98-1.01 | 0.451 |
| Observations | 248 | | | |
| Marginal *R^2^*/ Conditional *R^2^* | 0.311 / 0.324 | | | |

*Note.* ****p* < 0.001. Task Type was a categorical variable with two levels, “Free Play” (reference category) and “Elicitation.”

**Table S4.6**

*Output of GLMM Modelling Production of Joint Attention Bids, Elicitation Tests versus Free Play, with an Interaction between Age and Task Type, Age as Categorical Predictor*

|  | Joint Attention Bid Score | | | |
| --- | --- | --- | --- | --- |
| Predictors | Odds Ratios | SE | CI | *p* |
| (Intercept) | 0.08 | 0.06 | 0.02-0.36 | 0.001** |
| Age (7 months) | 1.00 | 1.05 | 0.13-7.77 | 1.000 |
| Age (8 months) | 3.69 | 3.24 | 0.66-20.65 | 0.137 |
| Age (9 months) | 9.38 | 7.95 | 1.78-49.39 | 0.008** |
| Age (10 months) | 8.44 | 7.19 | 1.88-51.96 | 0.012* |
| Task Type (Elicitation) | 7.92 | 6.73 | 1.50-41.89 | 0.015* |
| Age (7 months)* Task Type (Elicitation) | 1.65 | 1.97 | 0.16-17.16 | 0.677 |
| Age (8 months)* Task Type (Elicitation) | 1.08 | 1.16 | 0.13-8.80 | 0.942 |
| Age (9 months)* Task Type (Elicitation) | 0.29 | 0.30 | 0.04-2.20 | 0.232 |
| Age (10 months)* Task Type (Elicitation) | 0.92 | 1.01 | 0.11-7.89 | 0.941 |
| Observations | 248 | | | |
| Marginal *R^2^*/ Conditional *R^2^* | 0.328 / 0.349 | | | |

*Note.* **p* < 0.05, ***p* < 0.01. Task Type was a categorical variable with two levels, “Free Play” (reference category) and “Elicitation.”

**Table S4.7**

*Output of GLMM Modelling Production of Joint Attention Bids in each of the Elicitation Tests and Free Play, Age as Continuous Predictor*

|  | Joint Attention Bid Score | | | |
| --- | --- | --- | --- | --- |
| Predictors | Odds Ratios | SE | CI | *p* |
| (Intercept) | 0.30 | 0.07 | 0.19-0.47 | <0.001*** |
| Age (days, centred) | 1.01 | 0.00 | 1.01-1.02 | <0.001*** |
| Task Type (Interesting Sight) | 1.89 | 0.55 | 1.07-3.35 | 0.028* |
| Task Type (Interesting Sound) | 0.97 | 0.30 | 0.53-1.76 | 0.915 |
| Task Type (Moving Toy) | 1.65 | 0.48 | 0.93-2.93 | 0.084 |
| Observations | 493 | | | |
| Marginal *R^2^*/ Conditional *R^2^* | 0.098 / 0.129 | | | |

*Note.* **p* < 0.05, ***p* < 0.01, ****p* < 0.001. Task Type was a categorical variable with four levels, “Free Play” (reference category), “Interesting Sight”, “Interesting Sound” and “Moving Toy.”

**Table S4.8**

*Output of GLMM Modelling Production of Joint Attention Bids in each of the Elicitation Tests and Free Play, Age as Categorical Predictor*

|  | Joint Attention Bid Score | | | |
| --- | --- | --- | --- | --- |
| Predictors | Odds Ratios | SE | CI | *p* |
| (Intercept) | 0.15 | 0.05 | 0.08-0.29 | <0.001*** |
| Age (7 months) | 1.20 | 0.45 | 0.58-2.49 | 0.620 |
| Age (8 months) | 2.44 | 0.85 | 1.23-4.82 | 0.010* |
| Age (9 months) | 3.07 | 1.06 | 1.56-6.02 | 0.001** |
| Age (10 months) | 3.98 | 1.37 | 2.03-7.83 | <0.001*** |
| Task Type (Interesting Sight) | 1.89 | 0.55 | 1.07-3.35 | 0.028* |
| Task Type (Interesting Sound) | 0.97 | 0.30 | 0.53-1.76 | 0.915 |
| Task Type (Moving Toy) | 1.66 | 0.48 | 0.93-2.93 | 0.084 |
| Observations | 493 | | | |
| Marginal *R^2^*/ Conditional *R^2^* | 0.099 / 0.133 | | | |

*Note.* **p* < 0.05, ***p* < 0.01, ****p* < 0.001. Task Type was a categorical variable with four levels, “Free Play” (reference category), “Interesting Sight”, “Interesting Sound” and “Moving Toy.”

**Table S4.9**

*Output of GLMM Modelling Proportion of Tasks in which a Joint Attention Bid was Produced, at each Age Across all Three Elicitation Tests and Free Play Combined, Age as Continuous Predictor*

|  | Joint Attention Bid Score | | | |
| --- | --- | --- | --- | --- |
| Predictors | Odds Ratios | SE | CI | *p* |
| (Intercept) | 0.10 | 0.01 | 0.08-0.13 | <0.001*** |
| Age (days, centred) | 1.01 | 0.00 | 1.01-1.02 | <0.001*** |
| Observations | 124 | | | |
| Marginal R^2^ / Conditional R^2^ | 0.074 / 0.105 | | | |

*Note.* ****p* < 0.001.

**Table S4.10**

*Output of GLMM Modelling Proportion of Tasks in which a Joint Attention Bid was Produced, at each Age Across all Three Elicitation Tests and Free Play Combined, Age as Categorical Predictor*

|  | Joint Attention Bid Score | | | |
| --- | --- | --- | --- | --- |
| Predictors | Odds Ratios | SE | CI | *p* |
| (Intercept) | 0.05 | 0.01 | 0.03-0.09 | <0.001*** |
| Age (7 months) | 1.17 | 0.43 | 0.57-2.41 | 0.672 |
| Age (8 months) | 2.34 | 0.81 | 1.19-4.60 | 0.014* |
| Age (9 months) | 2.93 | 1.00 | 1.50-5.72 | 0.002** |
| Age (10 months) | 3.81 | 1.30 | 1.95-7.44 | <0.001*** |
| Observations | 124 | | | |
| Marginal R^2^ / Conditional R^2^ | 0.074 / 0.109 | | | |

*Note.* ****p* < 0.001, ***p* < 0.01, **p* < 0.05

**Supplementary Materials 5**

**Further Results**

The free play and elicitation tests (collapsed) were assessed in separate GLMMs that used the same model structure as those specified in the Results section. For the free play, the first model (Age, centred, as continuous fixed effect) revealed that Age was a significant predictor of Joint Attention Bid Production (log odds_baseline_=-1.70[-2.88, -0.94], *p*<0.001; log odds_age_=0.03[0.01,0.04], *p*<0.001). In the second model (Age as a categorical fixed effect), Tukey’s HSD post hoc tests on Age revealed significant increases in infants’ Joint Attention Bid production between 6 and 9 months (log odds = 2.84[0.15,5.53], *p*=0.033), and 7 and 9 months (log odds=2.84[0.15,5.53], *p*=0.033). However, in both cases the lower bound of the 95% confidence intervals was below the threshold of a small effect size. There was no significant difference between any two consecutive months.

For the elicitation tests, the first model (Age, centred, as continuous fixed effect) revealed that Age was a significant predictor of Joint Attention Bid Production (log odds_baseline_=0.56[0.15,1.03], *p*=0.007; log odds_age_=0.02[0.01,0.03], *p*=0.001). In the second model (Age as a categorical fixed effect), Tukey’s HSD post hoc tests on Age revealed significant increases in infants’ Joint Attention Bid production only between 6 and 10 months (log odds=2.06[0.14,3.97], *p*=0.028), again with the lower bound of the 95% confidence interval falling below the threshold of a small effect size. There was no significant difference between any two consecutive months.

**Table S5.1***Output of GLMM Modelling Production of Joint Attention Bids in Free Play, Age as Continuous Predictor*

|  | Joint Attention Bid Score | | | |
| --- | --- | --- | --- | --- |
| Predictors | Odds Ratios | SE | CI | *p* |
| (Intercept) | 0.18 | 0.08 | 0.07-0.45 | <0.001*** |
| Age (days, centred) | 1.03 | 0.01 | 1.01-1.04 | <0.001*** |
| Observations | 124 | | | |
| Marginal R^2^ / Conditional R^2^ | 0.204 / 0.483 | | | |

*Note.* ****p* < 0.001.

**Table S5.2***Output of GLMM Modelling Production of Joint Attention Bids in Free Play, Age as Categorical Predictor*

|  | Joint Attention Bid Score | | | |
| --- | --- | --- | --- | --- |
| Predictors | Odds Ratios | SE | CI | *p* |
| (Intercept) | 0.04 | 0.28 | 0.01-0.26 | 0.001*** |
| Age (7 months) | 1.00 | 1.11 | 0.11-8.74 | 1.000 |
| Age (8 months) | 4.84 | 4.67 | 0.73-32.13 | 0.103 |
| Age (9 months) | 17.08 | 16.86 | 2.47-118.17 | 0.004** |
| Age (10 months) | 14.33 | 14.04 | 2.10-97.78 | 0.007** |
| Observations | 124 | | | |
| Marginal R^2^ / Conditional R^2^ | 0.222 / 0.524 | | | |

*Note.* ***p* < 0.01. *** *p* < 0.001.

**Table S5.3***Output of GLMM Modelling Production of Joint Attention Bids across the Elicitation Tests, Age as Continuous Predictor*

|  | Joint Attention Bid Score | | | |
| --- | --- | --- | --- | --- |
| Predictors | Odds Ratios | SE | CI | *p* |
| (Intercept) | 1.75 | 0.37 | 1.16-2.64 | 0.008** |
| Age (days, centred) | 1.02 | 0.00 | 1.01-1.03 | 0.001** |
| Observations | 124 | | | |
| Marginal R^2^ / Conditional R^2^ | 0.119 / 0.141 | | | |

*Note.* ***p* < 0.01.

**Table S5.4**

*Output of GLMM Modelling Production of Joint Attention Bids across the Elicitation Tests, Age as Categorical Predictor*

|  | Joint Attention Bid Score | | | |
| --- | --- | --- | --- | --- |
| Predictors | Odds Ratios | SE | CI | *p* |
| (Intercept) | 0.66 | 0.28 | 0.29-1.50 | 0.322 |
| Age (7 months) | 1.65 | 0.96 | 0.53-5.14 | 0.390 |
| Age (8 months) | 4.00 | 2.48 | 1.18-13.49 | 0.026* |
| Age (9 months) | 2.74 | 1.63 | 0.85-8.81 | 0.091 |
| Age (10 months) | 7.82 | 5.49 | 1.98-30.93 | 0.003** |
| Observations | 124 | | | |
| Marginal R^2^ / Conditional R^2^ | 0.129 / 0.157 | | | |

*Note.* **p* < 0.05. ***p* < 0.01.

***SM5.1 Spontaneous Social Looks***

Figure S5.1 presents the developmental ordering of the scores received for each individual infant. It reveals that participants who received a score of “2” (for communicative joint attention bid) at 6 months did not necessarily continue to receive a score of “2” at every subsequent session. While 5 of the 25 infants (25%) received a score of “2” at every session, 11 of the 25 infants (44%) had at least one case in which they received a score of “2” at one month, and then did not receive a score of “2” (i.e. received a score of “1” for non-communicative look to E/M about the toy/stimulus) the following month. In all cases infants produced at least one additional joint attention bid after the first month in which they produced a bid (e.g., all participants who produced a joint attention bid at 6 months also produced a bid in at least one later month). Within each elicitation test, infants were only ever recorded producing a single Joint Attention Bid. Of the 31 free play sessions in which at least one joint attention bid was produced, infants produced one bid in 24 cases (77.42%, two bids in 6 cases (19.35%), and three bids in 1 case (3.23%).

**Figure S5.1**

*Individual Infants’ Highest Joint Attention Bid Score for Each Age, Collapsed Across Tasks*


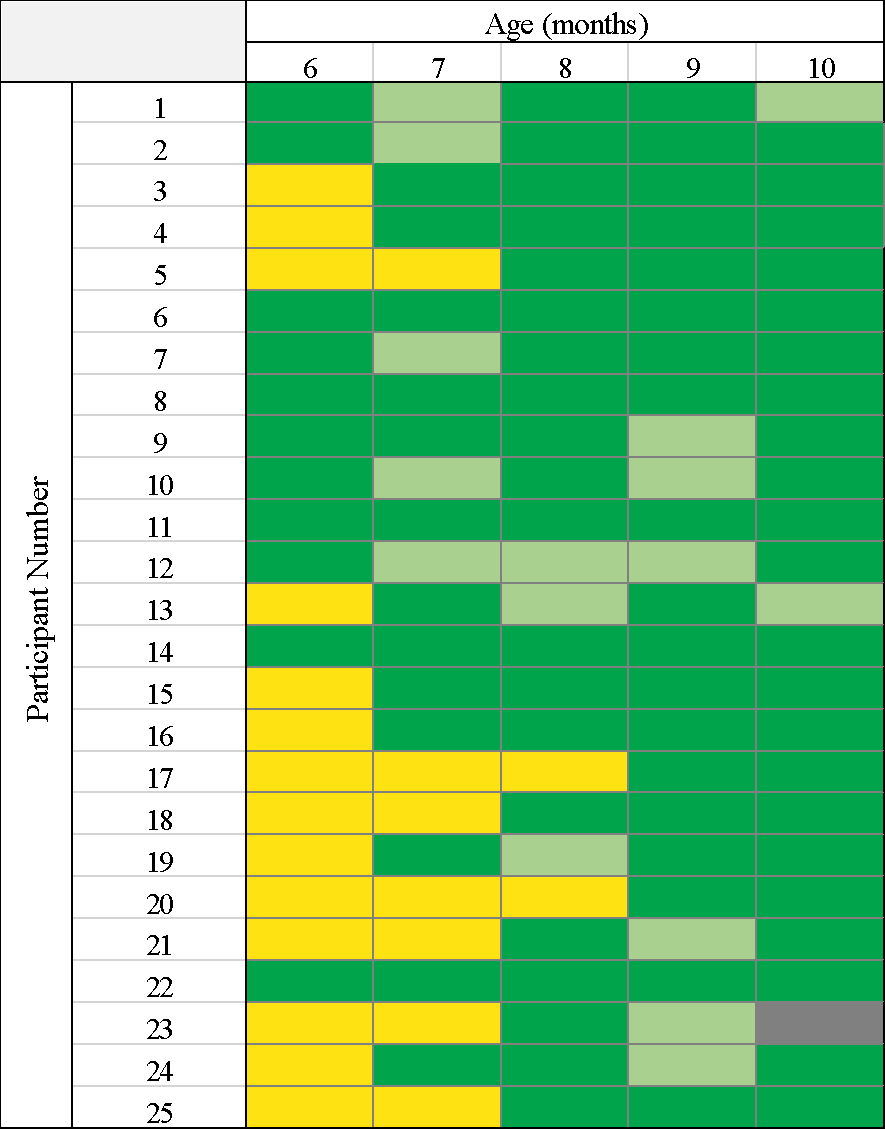


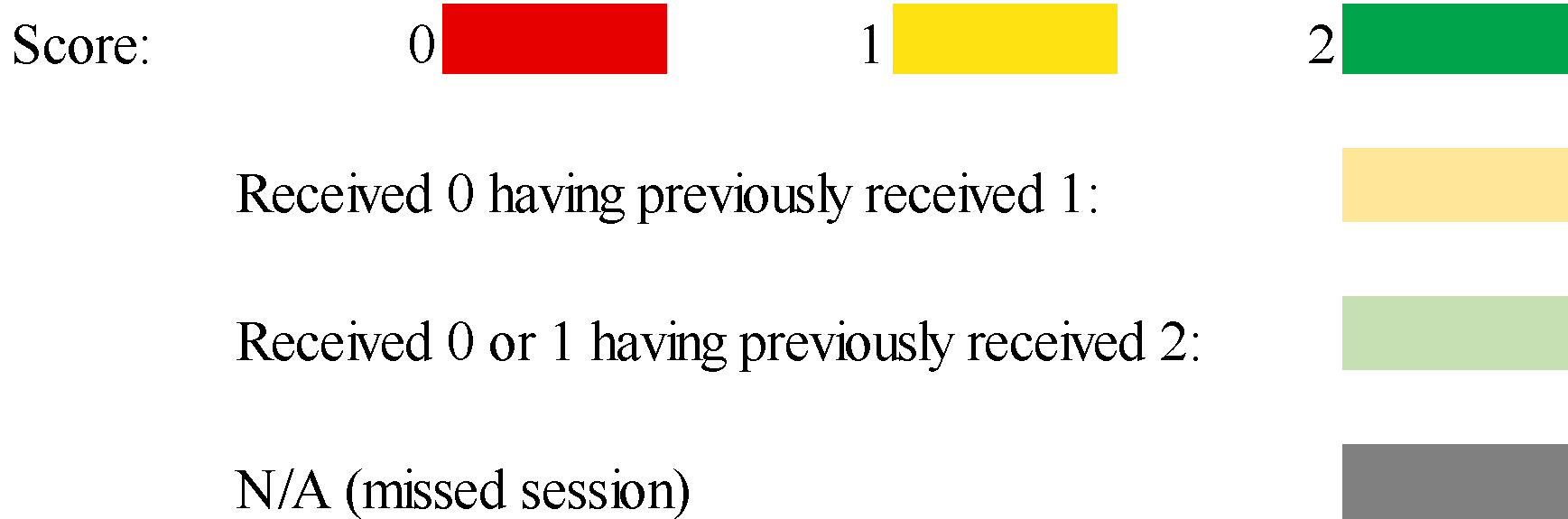


*Note.* For each participant at each session, the highest single score across all four tasks is used. Ages at which participants’ highest score was lower than a score they had received at a previous age are represented with a lighter shade. In all cases, participants who had previously received a “2” received at least a “1” in subsequent sessions.

Figure S5.2 shows the percentage of infants who received a “0”, “1”, or “2” as their highest score for each task at each age.

**Figure S5.2**

*Percentage of Infants who Received a Score of “0”, “1”, or “2” as their Highest Score for Joint Attention Bids at Each Age in Each Task*


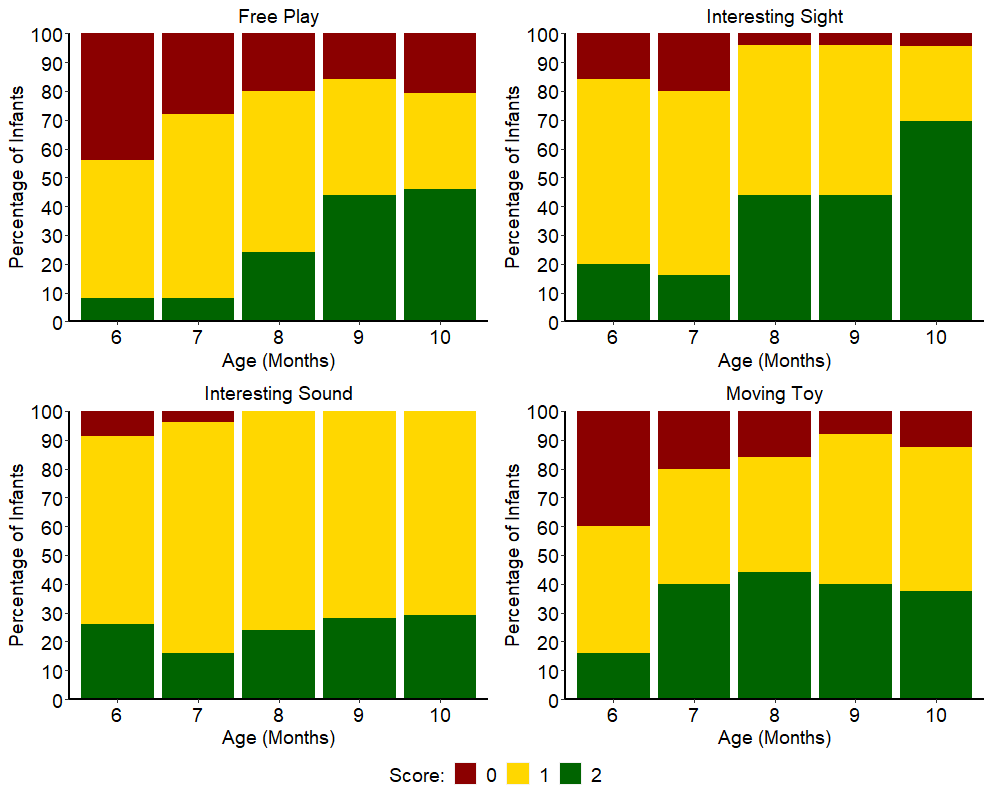


We also examined whether any particular tasks were more effective at eliciting spontaneous looks to E/M about the toy/stimulus, even if those looks were not necessarily communicative. We therefore conducted an analysis combining “1” and “2” scores into a “spontaneous social look” category. A GLMM was specified, with the dependent variable being spontaneous social look production (“0” = no spontaneous social look and “1” and “2” scores collapsed into a “spontaneous social look” score). Task Type (a categorical variable with each of the three Elicitation tests and free play as distinct categories) was included as a fixed effect. Tukey’s HSD post hoc tests on Task Type revealed that spontaneous social look production was significantly higher in the Interesting Sight test compared to the Free Play (log odds=1.25, *p*=0.005), in the Interesting Sound test compared to the Free Play (log odds=2.74, *p*<0.001), and in the Interesting Sound test compared to the Moving Toy test (log odds=2.33, *p*=0.001).

These results suggest that infants were especially likely to produce spontaneous social looks in the Interesting Sound test (though not any more than in the Interesting Sight test), and that infants were especially unlikely to produce spontaneous social looks in the Free Play (though not any less than in the Moving Toy test). This may have been in part because of the lack of an engrossing visual stimulus in the Interesting Sound test, which might have resulted in a greater likelihood of looking to the interaction partner. In contrast, the Moving Toy test (with a noisy, mobile object) and the Free Play (with the opportunity to manually and orally engage with objects) may have resulted in infants continuing to visually attend to the stimulus rather than breaking away from it to attend to the adult.

**Table S5.5**

*Output of GLMM Modelling Production of Spontaneous Social Looks in each of the Elicitation Tests and Free Play.*

|  | Spontaneous Social Look Score | | | |
| --- | --- | --- | --- | --- |
| Predictors | Odds Ratios | SE | CI | *p* |
| (Intercept) | 1.16 | 0.36 | 0.64-2.12 | 0.623 |
| Age (7 months) | 1.82 | 0.67 | 0.89-3.73 | 0.104 |
| Age (8 months) | 3.79 | 1.60 | 1.66-8.69 | 0.002** |
| Age (9 months) | 5.71 | 2.69 | 2.27-14.38 | <0.001*** |
| Age (10 months) | 4.05 | 1.77 | 1.72-9.52 | 0.001** |
| Task Type (Interesting Sight) | 3.51 | 1.34 | 1.67-7.40 | 0.001** |
| Task Type (Interesting Sound) | 15.41 | 9.69 | 4.49-52.85 | <0.001*** |
| Task Type (Moving Toy) | 1.50 | 0.48 | 0.80-2.81 | 0.206 |
| Observations | 494 | | | |
| Marginal *R^2^*/ Conditional *R^2^* | 0.310 / 0.325 | | | |

*Note.* ***p* < 0.01, ****p* < 0.001. Task Type was a categorical variable with four levels, “Free Play” (reference category), “Interesting Sight”, “Interesting Sound” and “Moving Toy.”

***SM5.2 Production of Joint Attention Bids, with and without Looks Back to the Stimulus***

Figure S5.3 illustrates that, in the tasks in which a look back could be coded (i.e., all the tasks with the exception of the Interesting Sound test), the majority of joint attention bids were produced with a look back to the stimulus at each age of testing.

**Figure S5.3**

*Infants’ production of Joint Attention Bids, with and without a Look Back to the Stimulus*


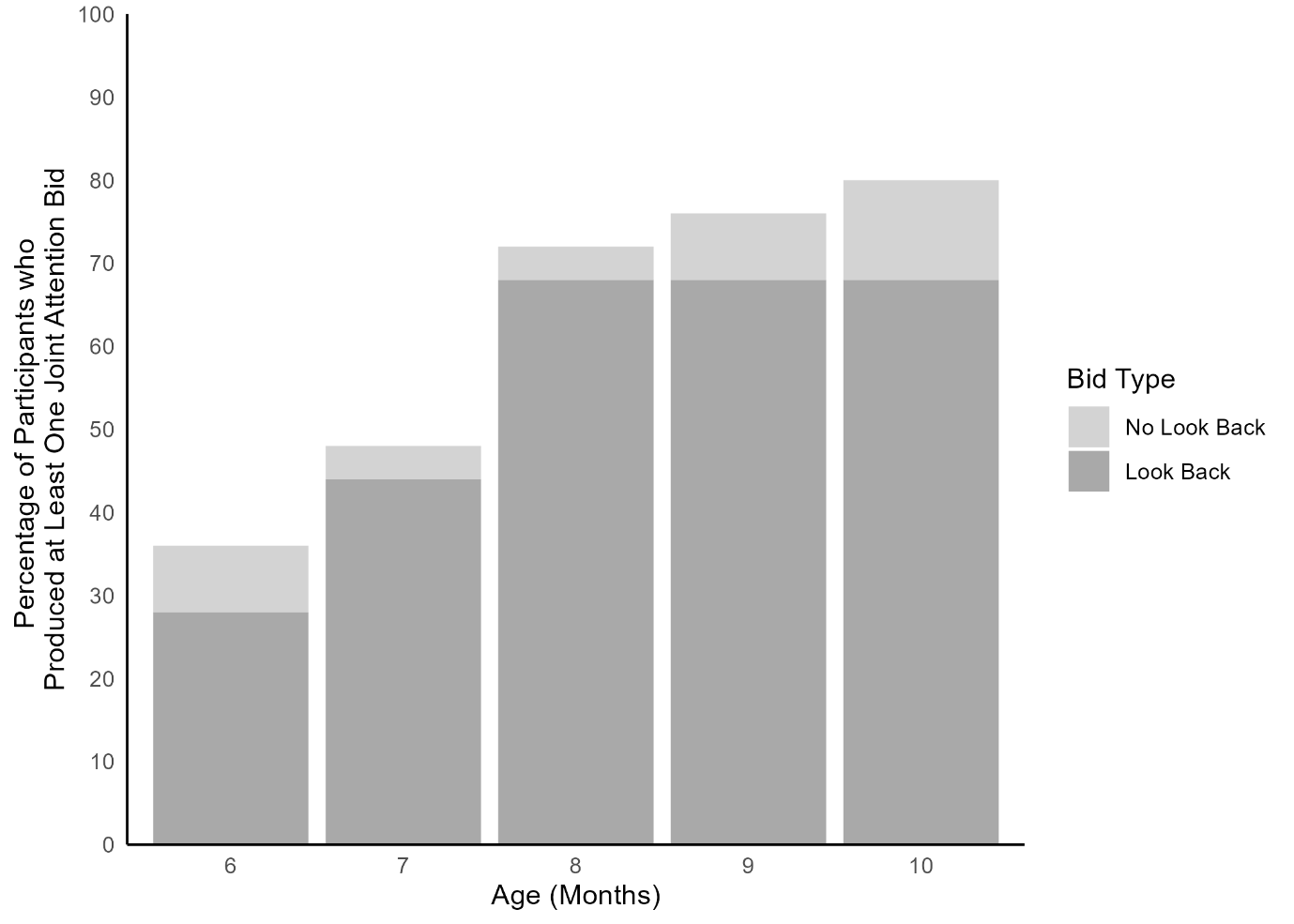


*Note.* Only data from the Interesting Sight test, Moving Toy test, and Free Play are included, as looks back to the stimulus could not be coded from the Interesting Sound test.

At 6 months, 28% of participants produced a joint attention bid with a look back; at 7 months it was 44%, and at 8, 9, and 10 months it was 68%.

**Supplementary Materials 6**

**R Package References**

Bates, D., Mächler, M., Bolker, B. & Walker, S. (2015). Fitting linear mixed-effects models using lme4. *Journal of Statistical Software*, *67*(1), 1–48. [doi:10.18637/jss.v067.i01](https://doi.org/10.18637/jss.v067.i01).

Gamer, M., Lemon, J., Fellows, I. & Singh, P. (2019). irr: Various coefficients of interrater reliability and agreement. R package version 0.84.1, https://CRAN.R-project.org/package=irr.

Lenth, R. (2024). emmeans: Estimated marginal means, aka least-squares means. R package version 1.10.0, https://CRAN.R-project.org/package=emmeans.

Lüdecke, D. (2023). sjPlot: Data visualization for statistics in social science. R package version 2.8.15, <https://CRAN.R-project.org/package=sjPlot>.

Lüdecke, D., Ben-Shachar, M., Patil, I., Waggoner, P., & Makowski, D. (2021). performance: An R package for assessment, comparison and testing of statistical models. *Journal of Open Source Software*, *6*(60), 3139. https://doi.org/doi:10.21105/joss.03139

Meyer, D., Zeileis, A., Hornik, K., Friendly, M. (2023). vcd: Visualizing Categorical Data. R package version 1.4-12, https://CRAN.R-project.org/package=vcd.

Müller, K. (2020). here: A simpler way to find your files. R package version 1.0.1, https://github.com/r-lib/here, <https://here.r-lib.org/>.

Wickham, H. (2007). Reshaping data with the reshape package. *Journal of Statistical Software*, *21*(12), 1–20. <https://doi.org/10.18637/jss.v021.i12>

Wickham, H., Averick, M., Bryan, J., Chang, W., McGowan, L. D. A., François, R., ... & Yutani, H. (2019). Welcome to the Tidyverse. Journal of Open Source Software, 4(43), 1686. https://doi.org/10.21105/joss.01686

**Supplementary Materials 7**

For the R Markdown script with full analyses, see <https://osf.io/z52ns/>, “ja_bid_markdown.html”.

**Supplementary Materials 8**

Data for main study analyses. Provided alongside the article, and via <https://osf.io/z52ns/>, “ja_bid_data.csv”.

**Supplementary Materials 9**

Data for supplementary analyses (see SM5.2). Provided alongside the article, and via <https://osf.io/z52ns/>, “ja_bid_look_back.csv”.

**Supplementary Materials 10**

Data for supplementary analyses (see R Markdown script in SM7, Part 9). Provided alongside the article, and via <https://osf.io/z52ns/>, “ja_bid_fp_bids.csv”.

**Supplementary Materials 11**

Data for reliability analyses. Provided alongside the article, and via <https://osf.io/z52ns/>, “ja_bid_reliability.csv”.
